# Electromagnetic Field‐Programmed Magnetic Vortex Nanodelivery System for Efficacious Cancer Therapy

**DOI:** 10.1002/advs.202100950

**Published:** 2021-07-18

**Authors:** Xiaoli Liu, Yifan Zhang, Yu Guo, Wangbo Jiao, Xiao Gao, Wee Siang Vincent Lee, Yanyun Wang, Xia Deng, Yuan He, Ju Jiao, Ce Zhang, Guoqing Hu, Xing‐Jie Liang, Haiming Fan

**Affiliations:** ^1^ Key Laboratory of Resource Biology and Biotechnology in Western China Ministry of Education School of Medicine Northwest University Xi'an Shaanxi 710069 China; ^2^ CAS Key Laboratory for Biomedical Effects of Nanomaterials and Nanosafety CAS Center for Excellence in Nanoscience National Center for Nanoscience and Technology of China No. 11, First North Road, Zhongguancun Beijing 100190 China; ^3^ University of Chinese Academy of Sciences No.19(A) Yuquan Road, Shijingshan District Beijing 100049 China; ^4^ Key Laboratory of Synthetic and Natural Functional Molecule Chemistry of the Ministry of Education College of Chemistry and Materials Science Northwest University Xi'an 710127 China; ^5^ Department of Engineering Mechanics Zhejiang University Hangzhou 310027 China; ^6^ Department of Materials Science and Engineering National University of Singapore Singapore 117573; ^7^ School of Life Sciences and Electron Microscopy Center of Lanzhou University Lanzhou University Lanzhou 730000 China; ^8^ Department of Nuclear Medicine The Third Affiliated Hospital of Sun Yat‐sen University 600 Tianhe Road Guangzhou Guangdong 510630 China; ^9^ State Key Laboratory of Cultivation Base for Photoelectric Technology and Functional Materials Laboratory of Optoelectronic Technology of Shaanxi Province National Center for International Research of Photoelectric Technology & Nanofunctional Materials and Application Institute of Photonics and Photon‐Technology Northwest University Xuefu Street No. 1 Xi'an 710127 China

**Keywords:** cancer therapy, electromagnetic field, intratumoral drug delivery, magnetic vortex nanovehicles, programmable magnetoresponsive activity

## Abstract

Effective delivery of anticancer drugs into the nucleus for pharmacological action is impeded by a series of intratumoral transport barriers. Despite the significant potential of magnetic nanovehicles in electromagnetic field (EF)‐activated drug delivery, modularizing a tandem magnetoresponsive activity in a one‐nanoparticle system to meet different requirements at both tissue and cellular levels remain highly challenging. Herein, a strategy is described by employing sequential EF frequencies in inducing a succession of magnetoresponses in the magnetic nanovehicles that aims to realize cascaded tissue penetration and nuclear accumulation. This nanovehicle features ferrimagnetic vortex‐domain iron oxide nanorings coated with a thermo‐responsive polyethylenimine copolymer (PI/FVIOs). It is shown that the programmed cascading of low frequency (L*f*)‐EF‐induced magnetophoresis and medium frequency (M*f*)‐EF‐stimulated magneto‐thermia can steer the Doxorubicin (DOX)‐PI/FVIOs to the deep tissue and subsequently trigger intracellular burst release of DOX for successful nuclear entry. By programming the order of different EF frequencies, it is demonstrated that first‐stage L*f*‐EF and subsequent M*f*‐EF operation enables DOX‐PI/FVIOs to effectively deliver 86.2% drug into the nucleus in vivo*. *This nanodelivery system empowers potent antitumoral activity in various models of intractable tumors, including DOX‐resistant MCF‐7 breast cancer cells, triple‐negative MDA‐MB‐231 breast cancer cells, and BxPC‐3 pancreatic cancer cells with poor permeability.

## Graphical abstract

A magnetic nano‐delivery system is developed to achieve drug nuclear delivery in vivo for efficient cancer therapy. By programming the order of different electromagnetic field (EF) frequencies, the first‐stage low frequency‐EF‐induced magnetophoresis and subsequent medium frequency ‐EF‐stimulated magneto‐thermia, enables drug‐carrying magnetic vortex nanorings to realize cascaded tissue penetration and drug nuclear accumulation in vivo. This nanodelivery system empowers potent antitumoral activity.

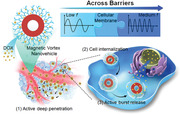

## 1 Introduction

Nanovehicles have emerged as a promising platform in delivering chemotherapeutic drugs to the tumor site for cancer therapy.^[^ ^1^, ^2^, ^3^, ^4^, ^5^, ^6^ ^]^ Despite the significant advances in the design of various nanovehicle‐based delivery systems,^[^ ^7^, ^8^, ^9^, ^10^, ^11^, ^12^ ^]^ the poor delivery efficiency of the chemotherapeutics to the final site of action, that is, the nucleus, has largely limited their antitumor efficacies. Such a limitation is due to the presence of a series of intratumoral transportation barriers at both the tissue and cellular levels.^[^ ^4^, ^13^, ^14^, ^15^, ^16^, ^17^, ^18^, ^19^ ^]^ When intravenously administered nanovehicles extravasate from the tumor blood vessels, they will encounter a series of obstacles. The interstitial extracellular matrix is the primary barrier that sterically prevents the diffusion of nanovehicles from the blood vessels into the tissue.^[^ ^2^, ^13^, ^20^, ^21^, ^22^, ^23^, ^24^, ^25^, ^26^ ^]^ In contrast to the passively diffusing nanovehicles, active drug delivery systems such as energy‐driven transcytosis and micro movable robots have shown promise in deep intratumoral penetration.^[^ ^20^, ^27^ ^]^ However, the successful arrival of the nanovehicle at the deep tumor site is only the first step. Bypassing the complex cellular membrane and nuclear envelope to transport drugs into the nucleus is the secondary challenge,^[^ ^28^, ^29^ ^]^ and this is particularly important for treating chemoresistant tumors. Although in vitro studies have shown the intracellular burst release of nanovehicles when triggered by exogenous stimuli can significantly enhance the drug accumulation in the nucleus,^[^ ^10^, ^30^, ^31^, ^32^, ^33^ ^]^ it remains a challenging task to integrate burst drug release with active tissue penetration for in vivo drug delivery. Therefore, to realize the superdeep delivery of chemotherapeutic drugs to the intra‐nuclear action site of the tumor cells, a nanovehicle delivery system that can facilitate on‐demand cascaded penetration and release across multi‐level biological barriers is urgently required.

The use of drug delivery systems based on magnetic nanovehicles has been the focus of biomedicine research. Magnetic nanovehicles possess significant potential in the realization of an electromagnetically activated superdeep drug delivery since electromagnetic fields (EFs) can penetrate through deep tissue, which, in turn, offers a remote and non‐invasive approach in deep tissue targeting. Furthermore, these magnetic nanovehicles can exhibit multiple magnetic‐responsive behaviors under different EF frequencies to produce various physicochemical effects. These physicochemical effects include magnetophoresis phenomenon at a low frequency (L*f*) gradient EF (≈30–500 Hz, L*f*‐EF)^[^ ^34^, ^35^, ^36^ ^]^ to facilitate the active transportation cargos across tumor interstitium, and magneto‐thermal effect at a medium frequency EF (≈100–500 kHz, M*f*‐EF)^[^ ^37^, ^38^, ^39^ ^]^ to trigger drug release on‐demand. This phenomenon exhibited by the magnetic nanovehicles under different EF frequencies presents an exciting opportunity in the modularized design and operation of an all‐in‐one drug delivery system, which can simultaneously attain efficient penetration and drug release on command.^[^ ^40^, ^41^ ^]^ Last but not least, both L*f*‐EF and M*f*‐EF are available in clinical settings with guaranteed safety.^[^ ^42^, ^43^ ^]^ Thus, it is possible to program these multiple magnetoresponsive functions in terms of space and time to steer the magnetic nanovehicles across intratumoral transportation barriers so as to achieve effective drug delivery. However, the concept of subjecting a magnetic nanovehicle to a programmable EF to achieve an in vivo all‐in‐one drug delivery system has yet to be materialized. This is partly due to the lack of suitable magnetic nanovehicles that can sufficiently trigger these magnetoresponsive actions. Superparamagnetic iron oxide nanoparticles (IONPs) are the main magnetic nanomedicine used in the (pre)clinic,^[^ ^44^, ^45^, ^46^ ^]^ but they are plagued with poor magneto‐responsive performances that can affect their drug delivery to the tumors. IONPs assembled in micro‐scale architecture can attain high magnetophoretic mobility that can realize tumor penetration.^[^ ^34^, ^40^, ^47^, ^48^ ^]^ However, this is often accompanied by a poor magneto‐thermal effect that results from the aggregation of IONPs^[^ ^49^ ^]^ and highly limited cell internalization,^[^ ^50^, ^51^ ^]^ which further hampers the intracellular drug burst release. Therefore, developing an optimal IONP‐based nanovehicle that is capable of efficient in vivo penetration driven magnetically and intracellular drug burst release in a one‐nanoparticle system is highly desirable for superdeep intratumoral drug delivery.

**Figure 1:**
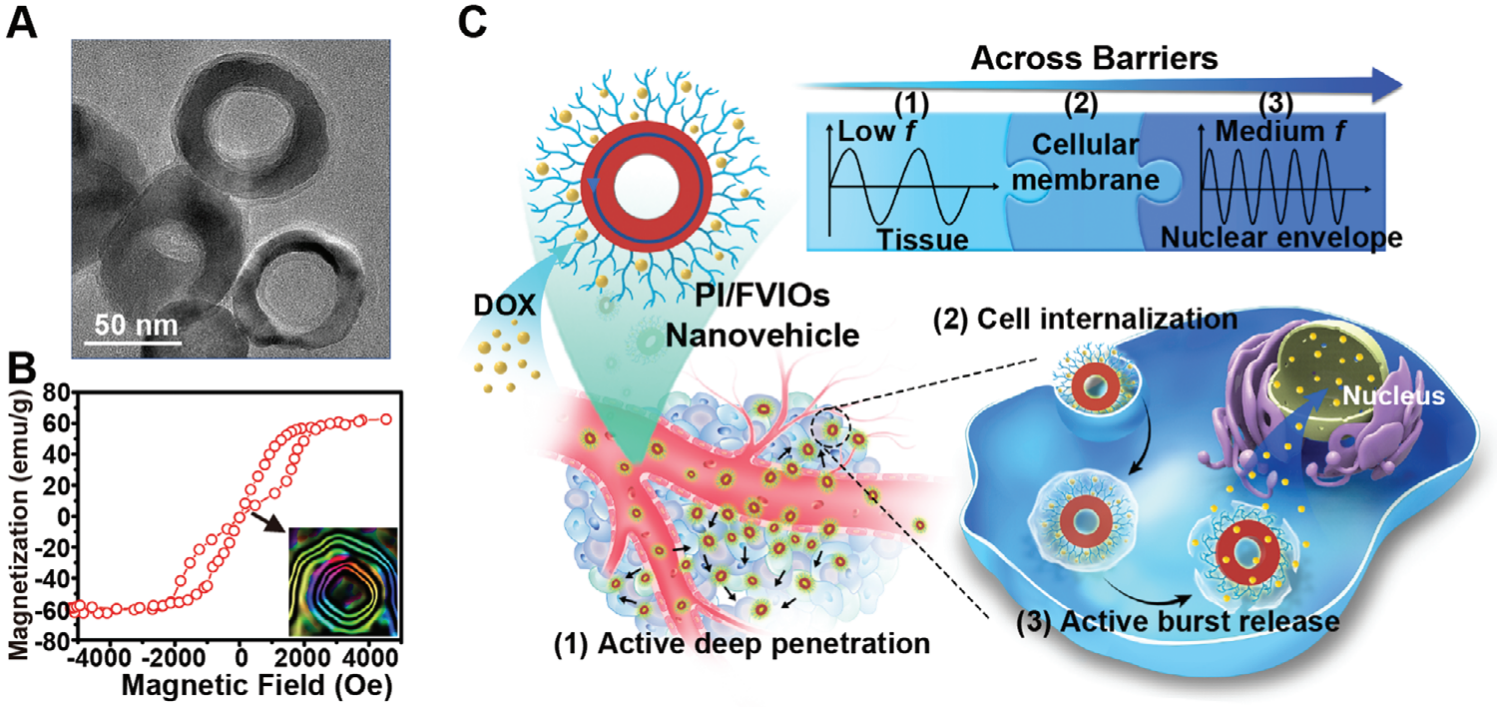
Design of magnetic drug delivery system based on switching EF frequency and programmable magnetoresponsive activity for intratumoral superdeep delivery. a) TEM image of DOX‐PI/FVIOs negatively stained with phosphotungstic acid to obtain sufficient contrast for the visualization of the surface coating layer. b) Magnetization curve of DOX‐PI/FVIOs measured with a VSM at room temperature. Inset: electron hologram of the PI/FVIOs. c) Illustration of the programmed EF actuation to overcome the intratumoral transportation barriers. The PI/FVIO nanovehicles possess an average outer diameter of ≈50 nm, and they feature a magnetic vortex domain structure that was coated with thermoresponsive PEI‐IBAm. 1) During the first stage, a low‐frequency EF endows the DOX‐PI/FVIOs with magnetophoretic mobility, and this process facilities the efficient penetration of the nanovehicle into the deep tumor tissue. 2) During the second stage, PI/FVIO nanovehicle, with its positive charge and appropriate size, is effectively internalized by the tumor cells. 3) During the third stage, a medium‐frequency EF stimulates the production of heat. The burst release of DOX from the thermo‐responsive nanovehicle results in the significant accumulation of DOX in the nucleus.

Herein, we propose a magnetic nanovehicle drug delivery system based on an EF frequency‐switch actuation in realizing cascade tissue penetration and nuclear accumulation for efficient cancer chemotherapy. The frequency of EF and the corresponding magneto‐responsive functions of the magnetic nanovehicle can be modulated to overcome various biological barriers in vivo. This magnetic nanovehicle features a 50 nm ferrimagnetic vortex iron oxide nanoring (FVIO)^[^ ^52^ ^]^ coated with a layer of thermo‐responsive polyethylenimine terminated with isobutyramide (PEI‐IBAm) groups (abbreviated as PI/FVIO, **Figure** **1**a). The size of FVIOs is specifically designed to balance the formation of the magnetic vortex structure and the physiological size requirement for their transportation in the tumor environment.^[^ ^53^, ^54^ ^]^ The unique ferrimagnetic vortex domain was confirmed by magnetic characterizations (Figure 1b). The results show that the PI/FVIOs possessed the critical parameters, for example, superior saturation magnetization and large hysteresis area with negligible remanence and coercivity, to meet the requirements for both high magneto‐mechanical and magneto‐thermal conversion efficiencies. The PEI‐IBAm copolymer^[^ ^40^, ^41^ ^]^ has a low critical solution temperature (LCST) of 48.6 °C (Figure S1, Supporting Information). This low LCST endows the PI/FVIO nanovehicles with a sensitive magnetothermal release behavior, and a positively charged surface to facilitate their uptake into the tumor cells efficiently. Doxorubicin (DOX) was used as a model drug since it possesses intrinsic fluorescence that can facilitate the in vivo image tracking of its biological fate. To prepare the DOX‐loaded PI/FVIOs (denoted as DOX‐PI/FVIOs; Figure S2 and Table S1, Supporting Information), PI/FVIOs were immersed in DOX HCl solution, whereby the DOX interacts with PI/FVIOs through noncovalent binding interactions (Figure S2, Supporting Information).^[^ ^55^, ^56^, ^57^ ^]^ We hypothesized that such a PI/FVIO nanovehicle can facilitate the delivery of DOX from the periphery of the tumor tissue to the nuclei of the cells deep in the tumor by executing programmable changes in the EF frequency, as described in Figure 1c. Upon exposure to an L*f*‐EF, the magnetophoretic mobility of DOX‐PI/FVIOs can lead to the active diffusion of nanovehicle through the tumor interstitial matrix (Barrier 1), and this results in its uniform distribution through the entire tumor tissue. After the arrival of DOX‐PI/FVIOs at the deep tumor site and their subsequent internalization across the cellular membrane (Barrier 2), a M*f*‐EF is applied to trigger an intracellular burst release of DOX. This sudden increase in the intracellular DOX concentration prompts the drug molecules to pass through the nuclear envelope (Barrier 3) and to ensure a sufficient drug concentration is present in the nucleus. In this study, we chose adriamycin‐resistant human MCF‐7 (MCF‐7/ADR) breast cancer as the model, and we demonstrated that delivering and maintaining a sufficient drug concentration in the nucleus is the pivotal determinant of the therapeutic efficacy of a treatment for the drug‐resistant tumor. The results show that this EF frequency‐programmed operation enabled a uniform distribution and sufficient nuclear accumulation of DOX throughout the tumor tissues. As a result, robust antitumor efficacy is achieved by employing this proposed magnetic drug delivery system.

## 2 Results and Discussion

### 2.1 L*f*‐EF Drives the Penetration of DOX‐PI/FVIOs Through the Tumor Interstitial Tissue

**Figure 2:**
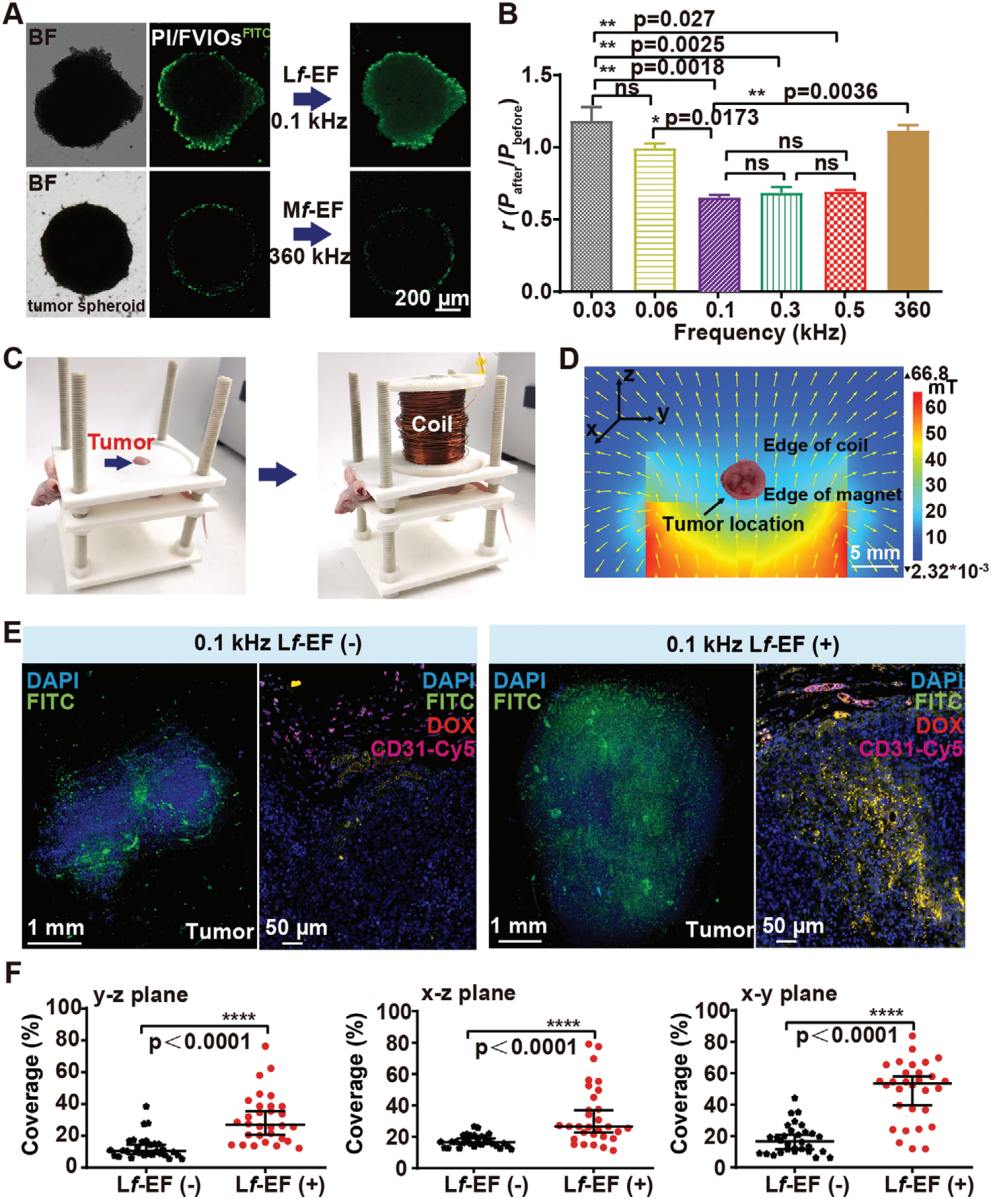
Enhanced tumor tissue penetration exhibited by DOX‐PI/FVIO nanovehicles under L*f*‐EF actuation. a) Fluorescence microscopy observations of the in vitro penetration of PI/FVIOs^FITC^ into MCF‐7/ADR breast tumor spheroids in the presence of L*f*‐EF (0.1 kHz) or M*f*‐EF (360 kHz). b) Quantification of the penetration capability of PI/FVIOs^FITC^ using the defined parameter *r* under EFs with various frequencies. Statistical analysis was conducted via one‐way analysis of variance (ANOVA) with Tukey's multiple comparison tests. c) (Left) A mouse bearing an MCF‐7/ADR xenograft tumor was held on a 3D‐printed PLA bed with the tumor exposed. (Right) The MCF‐7/ADR xenograft tumor was subjected to an L*f*‐EF. d) The cross‐sectional coil view of the magnetic flux distribution by COMSOL simulations in a finite element model. e) Average distribution of PI/FVIOs^FITC^ in a 3D reconfigured tumor (80 tumor sections). Fluorescent histology of the corresponding sections of the MCF‐7/ADR tumor, including the CD31‐Cy5‐stained blood vessel channel (purple), DOX channel (red), PI/FVIOs^FITC^ channel (green), and the DAPI‐stained nucleus channel (blue). f) Quantification of the tumor distributions of PI/FVIOs^FITC^ in each of the three tumor section planes. Data are presented as median ± interquartile range (n  =  30). Statistical analysis was conducted via a two‐tailed unpaired Student's *t*‐test. The *p* values < 0.05 were considered as statistically significant, and the range of *p* values was indicated by the number of asterisks (*), that is, *p < 0.05; **p < 0.01; ***p < 0.001; ****p < 0.0001.

To study the magnetophoretic mobility of PI/FVIOs in vitro, we used a model system consisting of MCF‐7/ADR breast tumor spheroids, which were prepared through the co‐culturing of 3T3 fibroblasts and MCF‐7/ADR cells in droplets.^[^ ^58^ ^]^ PI/FVIOs labeled with fluorescein isothiocyanate (PI/FVIOs^FITC^) were first adsorbed onto the outer layer of the spheroids by mixing, and they were subsequently exposed to L*f*‐EF for 10 min. We found that, under an L*f*‐EF of 0.1 kHz, PI/FVIOs^FITC^ could diffuse into the spheroid and they were eventually distributed throughout the entire spheroid (**Figure** **2**a, Figure S3, Supporting Information). In contrast, when the PI/FVIOs^FITC^ were subjected to a M*f*‐EF (360 kHz), these nanovehicles were only able to retain at the periphery of the spheroid. To quantify the frequency‐dependent diffusion ability of PI/FVIOs^FITC^ inside the tumor spheroid, we calculated the diffusion coefficient *r* (*r* = P_after_/P_before_, where P was used to analyze the distribution of PI/FVIOs^FITC^ in a spheroid before and after application of EF) (Figure S4, Supporting Information, details are provided in Section S1, Supporting Information). It is worth noting that the *r*‐value is inversely proportional to the magnetophoretic mobility. As shown in Figure 2b, when *f* < 0.1 kHz, the *r*‐value of PI/FVIOs^FITC^ decreases as *f* increases. The *r*‐value reached a minimum at *f* = 0.1 kHz, and remained low at *f* = 0.3 and 0.5 kHz. When *f* further increased to 360 kHz, there was a significant increase in the *r*‐value. Furthermore, the PI/FVIOs exhibited low mobility under a static magnetic field (details are provided in Movie S1, Supporting Information), which indicates the importance of an alternating magnetic field in actuating the diffusion of PI/FVIOs in the tumor spheroids.

We also performed numerical modeling of the frequency‐dependent penetration of PI/FVIOs (Figure S5, Supporting Information, details are provided in Section S2, Supporting Information). It is evident that PI/FVIOs could barely move in a static magnetic field and their displacements were small at *f* = 0.03 and 0.06 kHz. The transportation of PI/FVIOs was greatly enhanced by increasing the EF frequencies to 0.1 and 0.3 kHz. However, when the EF frequency increased to 0.5 kHz, there was a significant reduction in the movement of PI/FVIOs to the same level as for the static magnetic field. As such, these simulation results are in good agreement with the above experimental observation.

When L*f*‐EF was applied, the effects of alternating magnetic forces and the interaction with the surrounding substances were combined, which generated a random force on PI/FVIOs. The direction of this random force could change randomly with the same frequency as that of the EF. Thus, the random forces induced by the EF can disturb the motion of PI/FVIOs, increase the thermal dynamics, and reduce the chances of PI/FVIOs being trapped on the rough and sticky surfaces of the cells. A very low‐frequency magnetic field (0.03–0.06 kHz) is close to a static magnetic field, and too high a frequency (0.5 kHz) can lead to the leveling‐out of the disturbance by averaging over a short period of time. As a result, the best performance can be achieved by employing an optimal EF frequency, that is, 0.1–0.3 kHz.

We then evaluated the in vivo magneto‐actuated tumor‐penetrating effect of DOX‐PI/FVIOs in subcutaneous MCF‐7/ADR breast tumor‐bearing nude mice. PI/FVIOs^FITC^ ([Fe], 5 mg kg^−1^) were injected into the mice intravenously (i.v.). Dynamic magnetic resonance imaging (MRI) reveals that the maximum accumulation of DOX‐PI/FVIOs at the tumor site occurred at 6 h post‐injection (Figure S6, Supporting Information). At this time interval, the mice were placed in a polylactic acid (PLA) bed in a custom‐built magnetic device. Subsequently, the mice were exposed to L*f*‐EF for 10 min at the optimal frequency of 0.1 kHz (Figure 2c). The mice carrying MCF‐7/ADR xenograft tumors without L*f*‐EF treatment were included as a control group. The field distribution of L*f*‐EF, analyzed using electromagnetic simulations (Figure 2d) and experimental measurements (Figure S7a, Supporting Information), indicates a spatial magnetic gradient surrounding the tumor. After the L*f*‐EF treatment, the tumors were harvested, and they were embedded in paraffin. The tumor blood vessels were visualized using a Cy5‐tagged antibody against CD31 (purple) and the nuclei were stained with 4’,6‐diamidino‐2‐phenylindole (DAPI). The tumor tissue sections reveal that after the L*f*‐EF treatment at 0.1 kHz, DOX‐PI/FVIOs^FITC^ were distributed throughout the entire tumor tissue, and they were much farther away from the tumor vessels than the control without L*f*‐EF exposure (Figure 2e, Figure S7b, Supporting Information, details are provided in Movie S2, Supporting Information). The yellow color in the fluorescence images, which resulted from the overlay of DOX (red) and FITC‐labeled PI/FVIOs (green), indicates that DOX was still loaded inside the PI/FVIO nanovehicles after the treatment. In contrast, the signal detected from DOX‐PI/FVIOs^FITC^ in the control group was found mainly close to the blood vessels (details are provided in Movie S3, Supporting Information).

The penetration ability of PI/FVIO nanovehicles was quantified by analyzing the distribution of PI/FVIOs^FITC^ fluorescence signal in the solid tumor (Figure S7c, Supporting Information, and details are provided in Section S3, Supporting Information). Our results indicate that the tumor concentration of PI/FVIOs^FITC^ after L*f*‐EF actuation was ≈ 3 times higher than that of the control group (Figure 2f), which suggests that our strategy can overcome Barrier 1, that is, the stromal matrix. Thus, this result clearly demonstrates that the application of 0.1 kHz L*f*‐EF can lead to deep tumor penetration and uniform distribution of DOX‐PI/FVIOs inside the tumor.

### 2.2 M*f*‐EF Drives the Burst Release and Nuclear Accumulation of DOX

**Figure 3:**
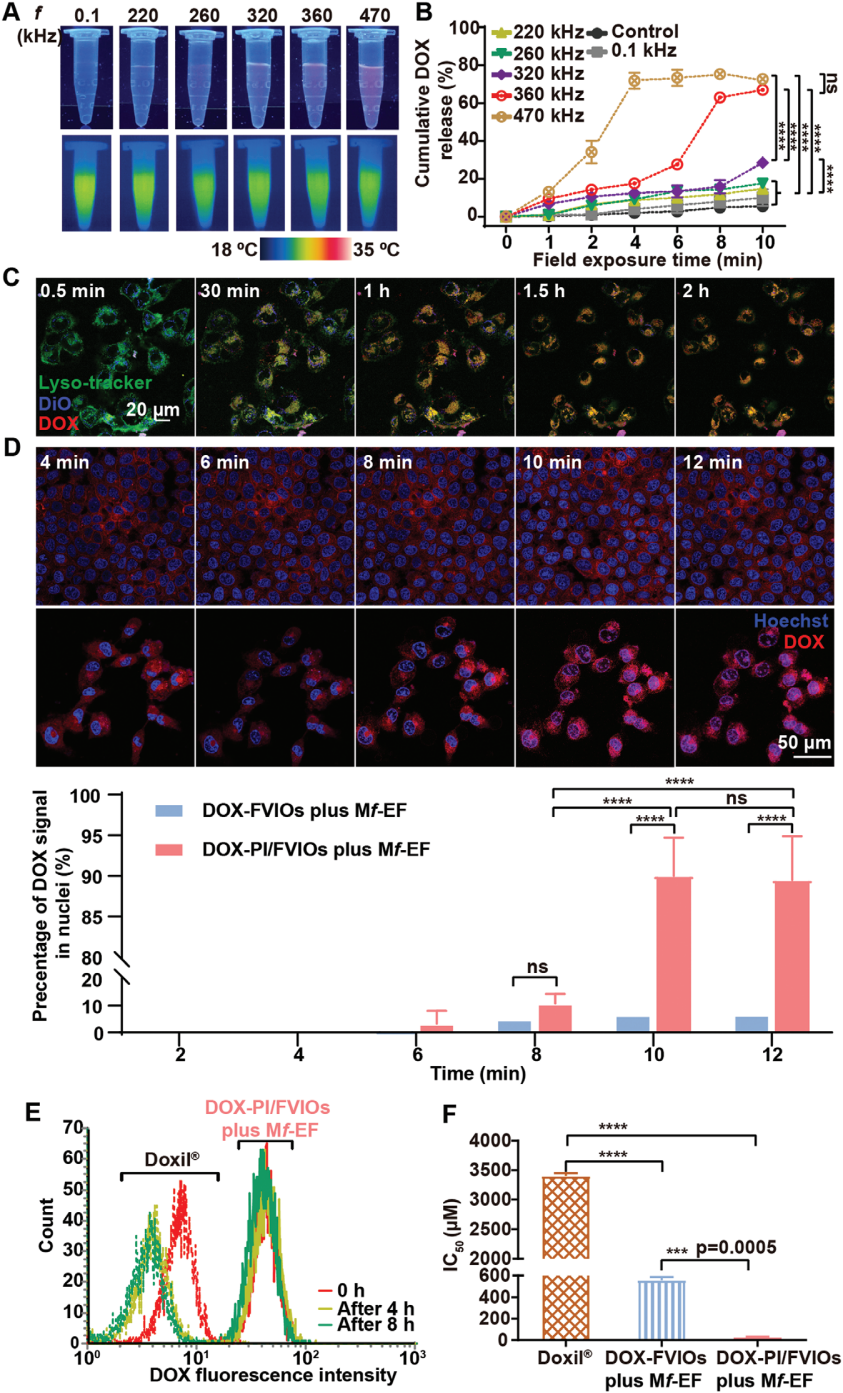
Enhanced burst release and nuclear accumulation of DOX after M*f*‐EF stimulus. a) Upper: Fluorescence images of DOX in the supernatant after the release from DOX‐PI/FVIOs in aqueous solution with EF exposure at various *f*. Lower: Thermal images of the same samples acquired by an infrared radiation (IR) camera. b) DOX release behavior of DOX‐PI/FVIOs during EF exposure at various *f*. Statistical analysis was conducted via two‐way ANOVA with Tukey's multiple comparison tests. c) Real‐time cell internalization of DOX‐PI/FVIOs in MCF‐7/ADR cells under a CLSM. The lysosomes were stained with LysoTracker, and the cytomembrane was labeled with DiO. d) Upper panel: CLSM images of real‐time DOX release from DOX‐FVIOs or DOX‐PI/FVIOs in MCF‐7/ADR cells after exposure to 360 kHz M*f*‐EF stimulus. Nuclei were stained with Hoechst. Lower panel: The percentage of DOX fluorescence in nuclei. Statistical analysis was conducted via two‐way ANOVA followed by Tukey's multiple comparison tests. e) Relative DOX fluorescence intensity in MCF‐7/ADR cells after pretreatment with Doxil or DOX‐PI/FVIOs under 10‐min Mf‐EF stimuli, followed by incubation in fresh medium for 4 or 8 h. f) IC_50_ of MCF‐7/ADR cells after different treatments. Statistical analysis was conducted via one‐way ANOVA with Tukey's multiple comparison tests. The *p* values < 0.05 were considered as statistically significant, and the range of *p* values was indicated by the number of asterisks (*), that is, *p < 0.05; **p < 0.01; ***p < 0.001; ****p < 0.0001.

We then investigated the release of DOX from DOX‐PI/FVIOs in vitro after the application of EFs at 0.1 kHz and between 220 to 470 kHz. To mimic the low concentration of nanomaterials in the animal tumor tissues, DOX‐PI/FVIOs at 50 µg mL^−1^ Fe were used in the experiments. A strong DOX fluorescence emission was only observed for those groups exposed to a M*f*‐EF in the frequency range of 320 to 470 kHz (**Figure** **3**a), which suggests the release of DOX from PI/FVIO nanovehicles within this range. Interestingly, the solution temperature remained at 26 °C during the release of DOX (Figure 3a), and this observation indicates that the PI/FVIO nanovehicles could achieve the magnetothermal induction of DOX release without affecting the temperature of the surrounding environment. The DOX release behavior of DOX‐PI/FVIOs (100 µg mL^−1^ Fe) was investigated by monitoring the absorption at 480 nm after the application of EFs at a frequency range of 0.1 to 470 kHz (Figure 3b). It can be observed that with the exposure to M*f*‐EF at 360–470 kHz for 10 min, a significant amount of DOX (up to ≈ 70%) was released. Meanwhile, less than 20% DOX was released after the treatment under *f* < 260 kHz. This phenomenon is ascribed to the magnetothermal induction of the drug release under M*f*‐EF at high frequency as the specific absorption rate (SAR) of PI/FVIOs is proportional to the *f* of the field (Figure S8, Supporting Information, details are provided in Section S4, Supporting Information). Moreover, a burst release of DOX was observed at 360 and 470 kHz EF (Figure 3b). Even though 470 kHz exposure induced a quick burst release of DOX at 4 min than that observed at 360 kHz, a similar cumulative DOX release (≈ 70%) was essentially observed for both 470 kHz and 360 kHz treatment at 10 min. Therefore, we chose to employ M*f*‐EF at 360 kHz for the following studies on the magnetothermal induction of DOX release.

To provide insights into the DOX delivery process by PI/FVIO nanovehicles, cell internalization was first studied. After co‐incubating DOX‐PI/FVIOs with MCF‐7/ADR cells, the fluorescences of DOX‐PI/FVIOs (red) and Lysotracker (green) were monitored using a confocal laser scanning microscope (CLSM) over time. After 2 h, a yellow fluorescence (which indicated a highly co‐localized of green and red) was observed (Figure 3c; Figure S9a, Supporting Information, and details are provided in Movie S4, Supporting Information). This result suggested the successful internalization of the nanovehicles for the delivery of DOX (red) into the cell endolysosomes. Following the endocytosis process (Figure S9b, Supporting Information), achieving a sufficient drug concentration in the nucleus is the last but most formidable hurdle in nuclear drug delivery. We used a CLSM to monitor the real‐time intracellular DOX distributions under a 360 kHz M*f*‐EF stimulus. As shown in Figure 3d and Movie S5, Supporting Information, there was a sharp increase in the DOX fluorescence in the nucleus after exposure to EF for 10 min. Additional CLSM images indicate the detachment of DOX from the PI/FVIO nanovehicles (Figure S9c, Supporting Information). To investigate the origin of the burst drug release, we have also synthesized non‐thermosensitive DOX‐FVIOs whereby the surface of FVIOs was coated with PEI without IBAm moiety (Figure S10, Supporting Information). This would allow the particles to still possess the same heat conversion capacity as the DOX‐PI/FVIOs (Figure S10, Supporting Information). When the cells were treated with DOX‐FVIOs under 360 kHz M*f*‐EF for 12 min, the DOX fluorescence remained in the perinuclear region (Figure 3d; Movie S6, Supporting Information), and the co‐localization of DOX and FVIO fluorescences suggests that DOX was in a loaded form (Figure S9c, Supporting Information). Further quantitative analysis indicates that 90% of DOX fluorescence was detected inside the nuclear regions for DOX‐PI/FVIOs after 10 min EF exposure. Such a result is more than 13‐fold higher than that observed for DOX‐FVIOs (Figure 3d; Figure S9d, Supporting Information). These data highlight the importance of the thermosensitive property of DOX‐PI/FVIOs in facilitating the burst release of the loaded drug for enhanced nuclear accumulation. Furthermore, the intracellular retention ability of these DOX‐PI/FVIOs after 360 kHz M*f*‐EF stimulus was studied. MCF‐7/ADR cells can decrease the intracellular drug concentration rapidly through drug efflux and/or drug metabolism. As indicated in Figure 3e, there was no change in the DOX signal from the treatment group of DOX‐PI/FVIOs subjected to 10‐min 360 kHz M*f*‐EF after 8h of further incubation, which clearly suggests its excellent intracellular retention of DOX. In stark contrast, for commercial Doxil treatment, there was a significant DOX signal decrease despite after 4 h incubation. It is clearly demonstrated that an efficient nuclear accumulation of DOX could be realized after subjecting the nanovehicles to M*f*‐EF, and this, in turn, can become a practical strategy in circumventing the MCF‐7/ADR cells.

Based on those results, the killing effect of DOX‐PI/FVIOs on MCF‐7/ADR cells under M*f*‐EF was investigated. The cell viability was neither affected by PI/FVIOs with nor without M*f*‐EF (360 kHz, 300 Oe, 10 min) (Figure S9e, Supporting Information), and this indicates that PI/FVIOs nanovehicles mediated magnetic hyperthermia itself did not produce a direct negative effect on the MCF‐7/ADR cells. As shown in Figure 3f, the half‐maximal inhibitory concentration (IC_50_) of DOX for Doxil against MCF‐7/ADR breast cancer cells was computed to be 3429 µm. However, for DOX‐PI/FVIOs, a significantly reduced IC_50_ of 26 µm was determined after exposure to 360 kHz M*f*‐EF treatment, which is approximately 130 times lower than that of Doxil (*p* < 0.0001). Such a result proves the enhanced anticancer effect of DOX with this system as compared to that with the Doxil. The same study was also conducted for DOX‐FVIOs containing the same concentration as that in the DOX‐PI/FVIOs. The IC_50_ for DOX‐FVIOs plus M*f*‐EF (same conditions as before) was 557 µm, which indicates the greater cytotoxicity of DOX‐PI/FVIOs than that of DOX‐FVIOs after M*f*‐EF stimulus.

### 2.3 Enhancing DOX Nuclear Delivery and Antitumor Efficacy by In Vivo Programmable EF Manipulation

**Figure 4:**
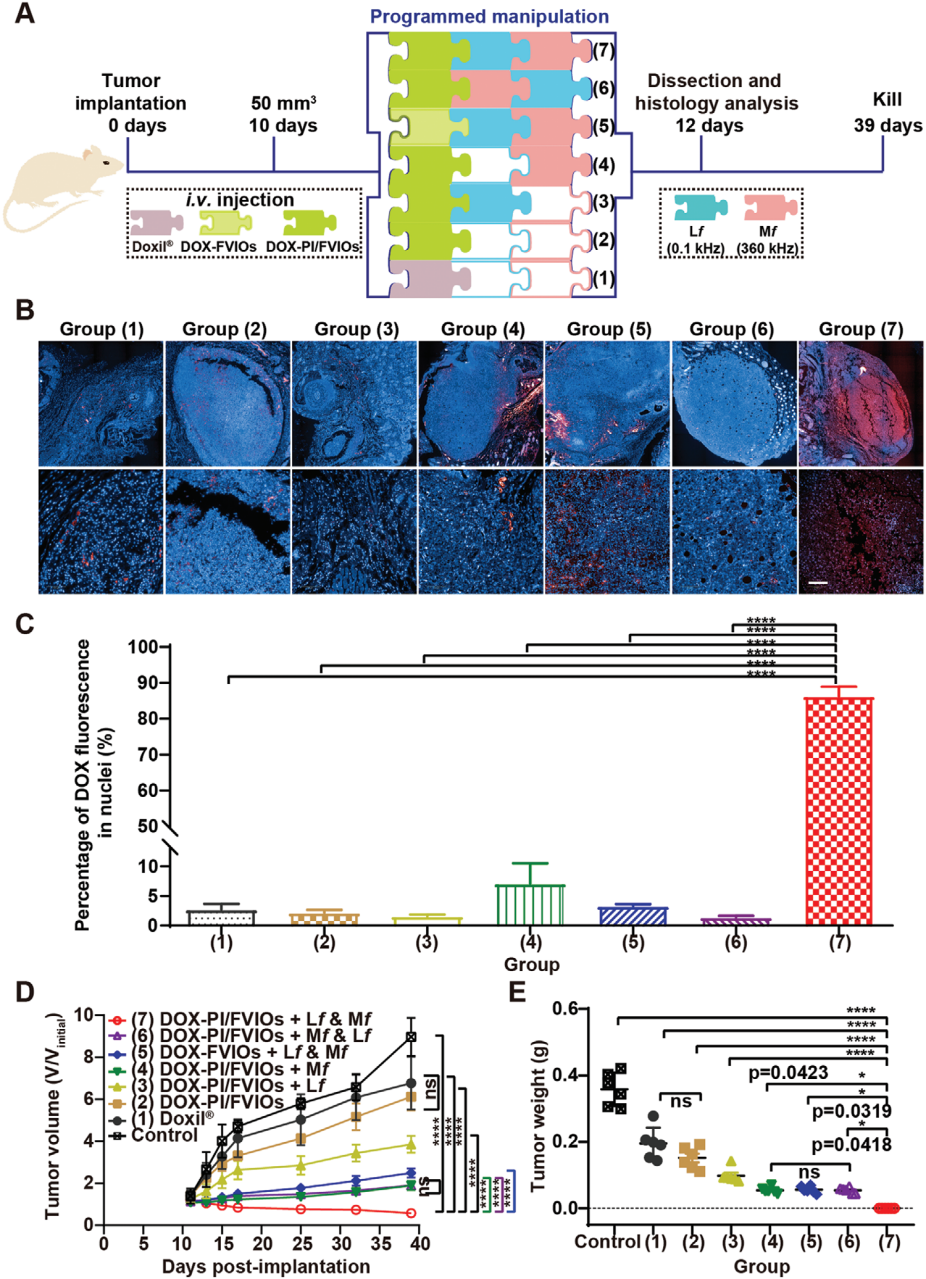
The in vivo programmable EF manipulation delivery system for superdeep drug delivery and its antitumor activity in MCF‐7/ADR tumors. a) Schematic illustration showing the design and treatment timeline of the programmable EF‐ manipulated delivery system. Nude mice bearing subcutaneous tumors derived from MCF‐7/ADR breast cancer cells (n = 6 mice per group) were intravenously administered with 100 µL of different samples at an equal DOX dose of 2.5 mg kg^−1^ body weight. Seven treatment groups were set up. Doxil, DOX‐FVIOs, or DOX‐PI/FVIOs were administered as a single i.v. injection. b) Fluorescence images of the tumor sections after various treatments. c) The percentage of DOX signal in the nuclei was then analyzed using HistoQuest system (TissueGnostics GmbH) according to the manufacturer's recommendations. ****p < 0.0001; statistical analysis was conducted via two‐way ANOVA followed by Tukey's multiple comparison post‐hoc test. d,e) The tumor volumes (d) and tumor weights (e) were measured. Two‐way ANOVA with Tukey's multiple comparison test was used to analyze differences between the groups. Data were reported as mean ± s.d. (n = 6). *p* values < 0.05 were considered as statistically significant. The *p‐*value range was indicated by the number of asterisks (*), that is, *0.01 < *p* < 0.05; **0.001 < *p* < 0.01; ***0.01 < *p* < 0.001; ****0.001 < *p* < 0.0001. ns, not significant (*p* > 0.05).

One of the most severe problems encountered in the clinical treatment of cancer is drug resistance. Drug‐resistant tumor cells can decrease the intracellular drug concentration rapidly through drug efflux and/or metabolism. Thus, achieving an effective drug concentration in the nucleus by mitigating the drug efflux is a pivotal determinant to the therapeutic efficacy of a treatment for the drug‐resistant tumor. Hence, we chose the subcutaneous MCF‐7/ADR xenograft tumor as a drug‐resistant tumor model to evaluate the in vivo nuclear delivery of DOX by the programmable EF‐manipulated delivery system (**Figure** **4**a). A biodistribution study of DOX‐PI/FVIOs i.v. injected into MCF‐7/ADR tumors‐bearing BALB/c nude mice was performed. After which, the distribution of DOX‐PI/FVIOs in the mice was quantified using ICP‐MS at different time intervals. As shown in Figure S11, Supporting Information, there was an effective accumulation of DOX‐PI/FVIOs in the tumor. The Fe content in the tumor reached the maximum value at 6–7 h, and then it decreased at 12–48 h. Six hours after i.v. injection of samples (with various formulations at an equivalent DOX dose of 2.5 mg kg^−1^ mouse), the MCF‐7/ADR tumor‐bearing BALB/c nude mice were exposed to a programmed EF‐actuation system. The commercial nanomedicine Doxil and non‐thermosensitive DOX‐FVIOs were also included as comparisons. The treatment groups were as follows: 1) Doxil; 2) DOX‐PI/FVIOs; 3) DOX‐PI/FVIOs plus L*f*‐EF; 4) DOX‐PI/FVIOs plus M*f*‐EF; 5) DOX‐FVIOs plus L*f*‐EF and M*f*‐EF treatment; 6) DOX‐PI/FVIOs plus M*f*‐EF and L*f*‐EF treatment; and 7) DOX‐PI/FVIOs plus L*f*‐EF and M*f*‐EF treatment. Subsequently, the tumors were harvested, prepared for paraffin sectioning, and then visualized using an Opera high content screening system (PerkinElmer). Figure 4b shows the even distribution of DOX inside the tumor and it mostly co‐localized with the nuclei for group 7, while most of the DOX delivered by the other treatments were confined at the periphery of the tumor. For group 5, a certain extent of DOX signals was observed inside the tumor, but they did not reside in the nucleus. Although hyperthermia can alter tumor physiology and it can relatively enhance the tumor penetration ability of nanomedicine,^[^ ^59^ ^]^ most DOX was still confined at the periphery of the tumor after exposing DOX‐PI/FVIOs to M*f*‐EF and subsequently to L*f*‐EF (group 6), which suggests that PI/FVIOs‐induced magnetic thermal effect exerted a negligible impact on the enhanced penetration. Collectively, the results demonstrate the importance of both the nanovehicle design and the order of EF treatment in achieving an efficient DOX nuclear delivery. The percentage of the DOX signal located in the nucleus was calculated to be 86.2% for group 7 (DOX‐PI/FVIOs plus L*f*‐EF and M*f*‐EF), which is more than an order of magnitude higher than those calculated for other groups (Figure 4c). This result suggests that DOX‐PI/FVIOs, after exposure to L*f*‐EF and subsequent M*f*‐EF, are the most effective system for the delivery of DOX to the cell nuclei in vivo.

The excellent nuclear delivery efficiency of DOX by DOX‐PI/FVIOs under the programmed EF operation urged us to assess the in vivo antitumor efficacy of our system. As monitored by an infrared thermal camera (Fotric 225), the macroscopic temperature at the tumor site remained at 37 °C for all EF‐exposure treated mice (Figure S12a, Supporting Information). Suppressed tumor growths to varying extents were observed for all treatments when compared to that observed for the control group (saline) (Figure 4d). Doxil or DOX‐PI/FVIOs exhibited limited abilities in tumor growth suppression, whereby the tumor size for each group increased 4.8 and 4.4‐folds after 39 days, respectively. The mice in the antitumor treatment group 4, that is, DOX‐PI/FVIOs plus M*f*‐EF, showed inhibited tumor growth. The mice after subjected to DOX‐PI/FVIOs plus L*f*‐EF and M*f*‐EF treatment showed the most significant tumor growth suppression. For this group, the tumors gradually shrank and almost disappeared. However, the antitumor activity in antitumor treatment group 5 (DOX‐FVIOs plus L*f*‐EF and M*f*‐EF) or group 6 (DOX‐PI/FVIOs plus M*f*‐EF and L*f*‐EF) was severely compromised when compared to that in group 7 (DOX‐PI/FVIOs plus L*f*‐EF and M*f*‐EF). Tumors from all treatment groups were excised and weighed at the end of day 39. Antitumor group 7 possessed the lowest tumor mass and the smallest excised tumors as compared to the other treatment groups, and this is consistent with the abovementioned results (Figure 4e and Figure S12b, Supporting Information).

The enhanced efficacy of the treatment can be attributed to the increased penetration ability of the nanovehicles into deeper tissue and the efficient nuclear delivery of DOX using a stepwise EF‐driven drug delivery system. None of the mice in any of the treatment groups showed significant changes in body weight during the entire treatment process (Figure S12c, Supporting Information). Histological studies were performed by examining the hematoxylin and eosin (H&E)‐stained tumor sections. The tumors of the control group consisted of densely packed cells, whereas those of antitumor group 7 contained abundant cells with extensive nuclear shrinkage and fragmentation (Figure S12d, Supporting Information). Additionally, the tumors were analyzed with deoxynucleotidyl‐transferase‐mediated nick end labeling (TUNEL) assay, which can assess the drug‐induced cell apoptosis by detecting the DNA fragments. The high TUNEL signal in the tumors from antitumor treatment group 7 as compared to those in other treatment groups (Figure S12d, Supporting Information) indicates the enhanced cell apoptosis in antitumor treatment group 7. This demonstration is remarkable as it validates the efficacy of the nanotechnologically driven, EF‐programmable drug delivery system on DOX‐resistant tumors.

**Figure 5:**
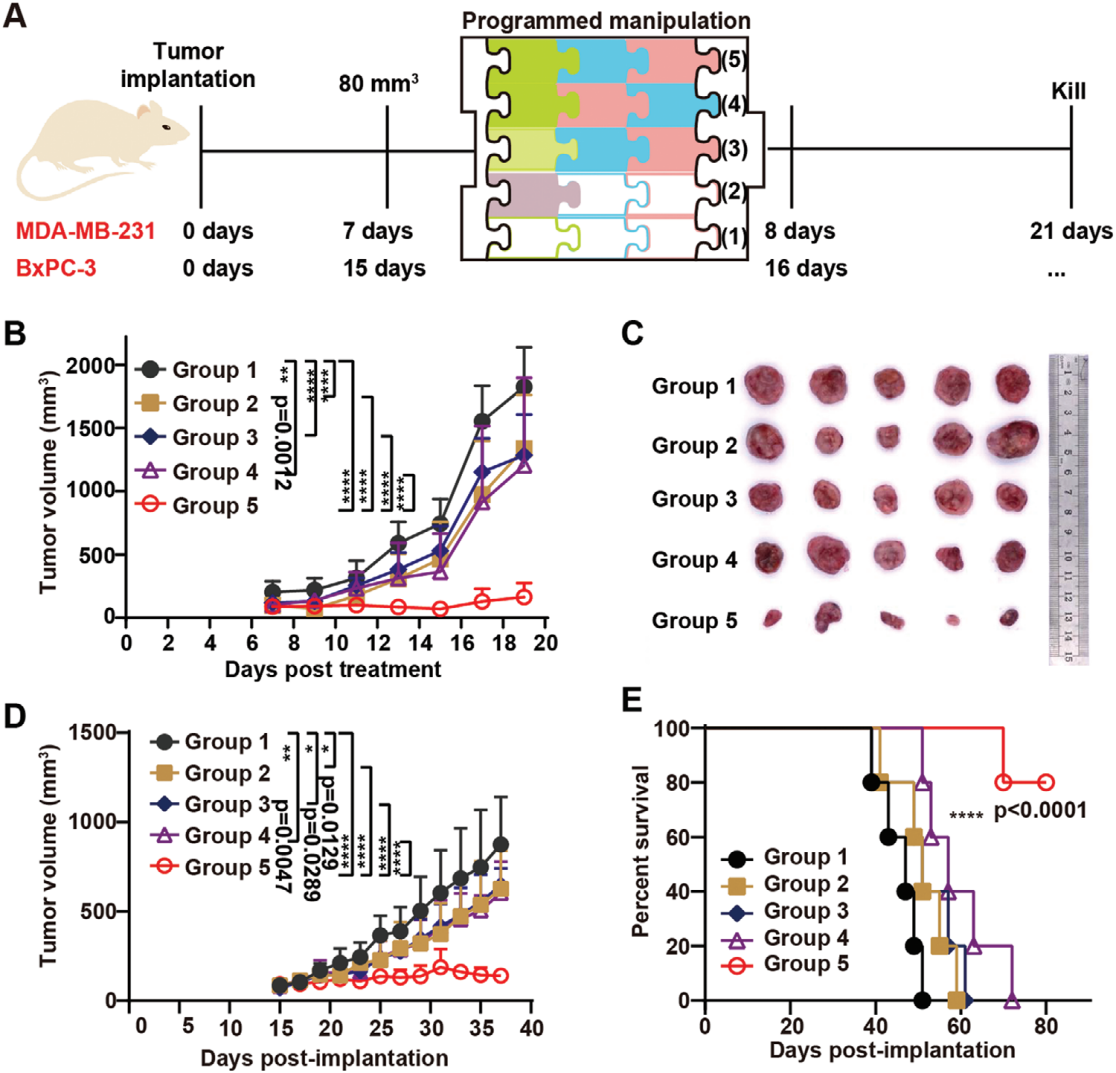
Antitumor activity of the programmable EF‐manipulated delivery system against triple‐negative breast MDA‐MB‐231 and pancreatic BxPC‐3 xenograft tumors. a) Schematic illustration of the treatment schedule. After the tumor transplantation, animals were randomly divided into 5 groups (n = 5 mice per group), and Doxil, DOX‐FVIOs, or DOX‐PI/FVIOs (2.5 mg kg^−1^ DOX per mouse body weight) were i.v. injected. One injection was given. Saline was used as a negative control. b) Effect of the different treatments on MDA‐MB‐231 tumor volumes. Data were reported as mean ± s.d. (n = 5). Statistical analysis was conducted via two‐way ANOVA with Tukey's multiple comparison test. c) Photographs of the MDA‐MB‐231 tumors on day 20. d) BxPC‐3 tumor volumes were measured. Tumor growth over time was compared by two‐way ANOVA with Tukey's multiple comparison test. Data were reported as mean ± s.d. (n = 5). *p* values < 0.05 were considered as statistically significant. The range of *p* values was indicated by the number of asterisks (*), that is, *0.01 < *p* < 0.05; **0.001 < *p* < 0.01; ***0.01 < *p* < 0.001; ****0.001 < *p* < 0.0001. e) Kaplan‐Meier survival curves of BxPC‐3 tumor‐bearing BALB/c nude mice in different treatment groups. The statistical significance of the survival analysis in (e) was calculated by the log‐rank (Mantel‐Cox) test.

To further demonstrate the versatility of this DOX‐PI/FVIOs‐based drug delivery system, we utilized a triple‐negative MDA‐MB‐231 breast tumor model to evaluate its in vivo therapeutic efficacy (**Figure** **5**a). Five antitumor treatment groups were set up as follows: 1) Saline; 2) Doxil; 3) DOX‐FVIOs plus L*f*‐EF and M*f*‐EF treatment; 4) DOX‐PI/FVIOs plus M*f*‐EF and L*f*‐EF treatment; and 5) DOX‐PI/FVIOs plus L*f*‐EF and M*f*‐EF treatment. As shown in Figure 5b, Doxil (group 2), DOX‐FVIOs + L*f*‐EF & M*f*‐EF (group 3), and DOX‐PI/FVIOs + M*f*‐EF & L*f*‐EF (group 4) exhibited modest antitumor efficacies as compared to the control group treated with physiological saline. However, the mice receiving DOX‐PI/FVIOs plus L*f*‐EF then M*f*‐EF (group 5) showed a 94% tumor inhibition rate. Furthermore, as shown in the photographs in Figure 5c and Figure S13a, Supporting Information, the tumors collected at the end of treatment demonstrated the same trend. Moreover, this DOX‐PI/FVIOs‐based drug delivery system triggered little systemic toxicity as revealed by the stable bodyweight of the mice (as depicted in Figure S13b, Supporting Information).

Finally, the antitumor activities were also assessed in the subcutaneous pancreatic BxPC‐3 tumors. Pancreatic BxPC‐3 tumors are difficult to treat due to their rich dense connective tissue and protective dense stroma, which greatly limit the efficiency of the drug delivery process. Five treatment groups were set up, as described above for the MDA‐MB‐231 model. Figure 5d shows that the tumors in group 5, that is, a single injection of DOX‐PI/FVIOs plus L*f*‐EF then M*f*‐EF, were much smaller with an inhibition rate of 84% by day 37 than other treatment groups. It should be noted that all mice in the saline group died by day 51 (Figure 5e). However, 80% of the mice in treatment group 5 were still alive at day 80 (Figure S13c, Supporting Information). During the entire treatment period, none of the mice showed significant changes in body weight (Figure S13d, Supporting Information). According to the H&E staining, immunohistochemical staining, and TUNEL analysis of the tumor sections, extensive apoptosis in the tumors from treatment group 5 was observed (Figure S13e, Supporting Information).

One of the major concerns associated with the clinical translation of a functional nanomedicine is the cytotoxicity that arises from the nonspecific accumulation of the nanomedicine in healthy organs. Here, a comprehensive in vivo safety assessment of the DOX‐PI/FVIOs was performed. Non‐tumor‐bearing healthy Sprague Dawley (SD) rats were injected with DOX‐PI/FVIOs and the major organs, that is, brain, heart, kidney, liver, lung, and spleen, were harvested for histological analysis. No major organ damage was observed in the mice on day 30 post‐injection of DOX‐PI/FVIOs (Figure S14, Supporting Information), which indicates that DOX‐PI/FVIOs possessed good biocompatibility. No significant changes were observed in the liver function and blood chemistry after treating the SD rats with DOX‐PI/FVIOs at a dose of 5 mg kg^−1^ Fe (Figure S15, Supporting Information).

In summary, a remote field‐programmable DOX‐PI/FVIO nanovehicle was developed in this work to achieve superdeep nuclear delivery of DOX for efficient cancer treatment. Cascading magnetoresponsive activities were observed for DOX‐PI/FVIOs, whereby it involved an L*f*‐EF‐induced magnetomechanical force to facilitate the diffusion of DOX towards the deeper tumor tissue, and a M*f*‐EF‐stimulated nanoheating to trigger the burst release of DOX for successful nuclear entry. When compared to the previous studies, our current proposed strategy has several unique features. First, the previous delivery systems focused solely on tumor penetration^[^ ^41^, ^60^ ^]^ or on‐demand drug release.^[^ ^33^, ^61^, ^62^ ^]^ However, our system is devised based on a one‐nanoparticle system that can simultaneously achieve efficient in vivo penetration that is driven magnetically and intracellular drug burst release. Second, the penetration of magnetic nanocarriers into the tumor by magnetic field reported in previous studies remains to be less‐than‐satisfactory. This is largely due to the poor penetration of magnetic nanocarrier into the tumor under static magnetic field that was employed in these studies.^[^ ^40^, ^41^, ^63^ ^]^ For the first time, we have demonstrated, using both numerical modeling and experimental studies, that low‐frequency EF (alternating magnetic field) can be more effective in inducing the PI/FVIOs penetration as compared to that under a static magnetic field (Movie S1, Supporting Information). Third, the treatment of drug‐resistant tumors presents a tremendous challenge for the state‐of‐art anticancer treatment. This is largely due to the poor accumulation and retention of drugs in the nucleus. Thus, even though previous works have demonstrated the capability of magnetic hyperthermia in triggering drug release,^[^ ^62^, ^64^ ^]^ these strategies remain to be ineffective in the treatment of drug‐resistant tumors. As demonstrated in this work, our nanoplatform was able to exhibit an extremely high localized temperature under M*f* EF, which could facilitate the nucleus accumulation and retention of DOX. Consequently, the superior antitumor activities of this strategy were successfully demonstrated in DOX‐resistant MCF‐7 breast tumor, aggressive triple‐negative MDA‐MB‐231 breast tumor, and BxPC‐3 pancreatic tumor with poor permeability. This result implies the broad applicability of this current strategy when compared to the previously reported delivery systems that relied on tumor microenvironments stimulus, for example, mild acidity, redox potential, and high‐level enzymes.

## 3 Conclusion

We have developed a programmable magnetic drug delivery system by switching EF frequency to achieve superdeep nuclear delivery for efficient cancer chemotherapy. The DOX‐PI/FVIOs exhibit cascaded magnetoresponsive activity, including an L*f*‐EF‐induced magnetomechanical force to facilitate drug diffusion towards the deeper tumor tissue, and M*f*‐EF‐stimulated nanoheating to trigger a burst release of DOX for successful nuclear entry. Results in three models of intractable solid tumors, including DOX‐resistant MCF‐7 breast cancer, aggressive triple‐negative MDA‐MB‐231 breast cancer, and poorly permeable BxPC‐3 pancreatic cancer, demonstrated the significantly improved antitumor efficacy of DOX‐PI/FVIOs compared to the control treatments. These results suggest that this innovative nanovehicle delivery system can overcome major delivery barriers. Although DOX is used as the model chemotherapeutic here, this magnetic drug delivery strategy could be applied for the delivery of other insoluble drugs to treat a variety of diseases. Our design strategy may open up a new avenue for developing the next generation of nanovehicle‐based delivery systems.

## 4 Experimental Section

### Chemicals

Absolute ethanol, absolute methanol, chloroform, hexane, triethylamine, ferric chloride hexahydrate (>99.0%), ammonium dihydrogen phosphate, sodium chloride, and N,N‐dimethylformamide were purchased from Kelong Chemical Co. Ltd, (Chengdu, China), and they were used as received. Trioctylamine (98%), oleic acid (90%), isobutyric anhydride, and hyperbranched polyethylenimine (*M*_n_ = 1800 Da) were purchased from Aladdin Industrial Corporation. Doxorubicin hydrochloride was purchased from Sigma‐Aldrich. Anhydrous potassium carbonate (99%) was purchased from Tianjin Hongyan Reagent Factory. FITC (fluorescein isothiocyanate isomer I, ≥90%) was purchased from Sigma‐Aldrich.

### Synthesis of FVIOs

FVIOs with an outer diameter of 50 nm were prepared via a thermal transformation of *α*‐Fe_2_O_3_ nanorings. *α*‐Fe_2_O_3_ nanorings were prepared via a modified synthesis process according to the previously reported method.^[^ ^52^ ^]^ 0.8 mL aqueous FeCl_3_ solution (0.5 m), 0.72 mL aqueous NH_4_H_2_PO_4_ solution (0.02 m), and 0.72 mL aqueous NaCl solution (0.02 m) were first mixed under vigorous stirring. Deionized water (DI water) was then added to the mixed solution to reach a final volume of 40 mL, and the solution was later stirred for another 10 min. Subsequently, the mixture was transferred into a Teflon‐lined stainless‐steel autoclave with a capacity of 50 mL. After which, it was subjected to hydrothermal treatment at 220 °C for 38 h. The autoclave was allowed to cool down to room temperature naturally. The red precipitate was collected after centrifugation, and it was washed with DI water and absolute ethanol repeatedly. The precipitate was later dried under vacuum at 80 °C for 24 h. 0.5 g as‐obtained *α*‐Fe_2_O_3_ nanorings were then annealed at 450 °C for 2 h under a constant 5% H_2_/95% Ar flow at 200 sccm in a tube furnace to finally obtain the FVIOs.

### Synthesis of PI

The hyperbranched polyethylenimine (*M*_w_ = 1800 Da) with terminal isobutyric‐amide groups (PEI‐IBAm, abbreviated as PI) was prepared according to the literature.^[^ ^65^, ^66^, ^67^ ^]^ In brief, 8.2 g isobutyric anhydride was added dropwise to 50 mL chloroform solution that comprised of 3.1 g PEI 1.8 k and 5.6 g triethylamine with vigorous stirring in an ice bath under an argon atmosphere. Subsequently, the reaction was carried out at room temperature for 24 h. Then, the temperature was raised to 65 °C for 2 h to complete the reaction. After cooling down to room temperature, the as‐produced salt was filtered off. The filtrate was washed with 10% potassium carbonate solution until a basic aqueous solution was achieved. Next, it was washed with saturated brine until a neutral pH was obtained. After removing the organic volatiles under vacuum, the obtained residue was then dissolved in 35 mL methanol. To remove the low molecular weight impurities, dialysis was performed against methanol for 2 days with the use of benzoylated cellulose membrane (molecular weight cut off = 1000 g mol^−1^). Finally, the methanol solvent was removed under vacuum, and the product was dried under vacuum for 24 h.

### Synthesis of PI/FVIOs

20 mg FVIOs powder was uniformly dispersed in a mixture of 0.4 mL oleic acid and 10 g trioctylamine using ultrasonication. Then the mixture was heated to 280 °C in an Ar environment with continuous stirring for 40 min. After the mixture cooled down, the resulting oleic acid‐coated FVIOs were collected by centrifugation at 8000 rpm for 10 min, and it was subsequently washed several times with ethanol/hexane. The purified oleic acid‐coated FVIOs were then re‐dispersed in chloroform.

PI/FVIOs were synthesized by modifying the surface of oleic acid‐coated FVIOs with PI via a ligand exchange reaction. The oleic acid‐coated FVIOs were dispersed in chloroform/DMF (in a ratio of 2:3, and a total volume of 10 mL) with a concentration of 1 mg mL^−1^ to form Mixture A. After that, 200 mg PI was dissolved in chloroform/DMF (in a ratio of 1:1, and a total volume of 6 mL) to form Mixture B. Then, Mixture B was added dropwise into Mixture A, and the mixture was shaken overnight at 50 °C. After that, the sample was collected by centrifugation at 8000 rpm for 10 min, and then the collected sample was washed three times with DI water.

For the synthesis of PEI/FVIOs, 200 mg PEI was mixed with 10 mg oleic acid‐coated FVIOs in 20 mL chloroform, and the mixture was shaken for 4 h under ambient conditions. Then, the solvent was evaporated under an Ar atmosphere, and the dried nanoparticles were re‐dispersed into the water. Further centrifugation (8000 rpm, 10 min) was conducted to remove the unbound PEI polymer that was present in the supernatant.

### Synthesis of DOX‐PI/FVIOs

For the further preparation of DOX‐PI/FVIOs, 1 mg PI/FVIOs was mixed with 5 mL DOX HCl solution (1 mg mL^−1^) in phosphate buffer solution (PBS). After stirring for 24 h under dark conditions, DOX‐PI/FVIOs were collected by centrifugation. The DOX content was determined using UV–vis measurements at the wavelength of 480 nm. The same procedure was used in the synthesis of DOX‐FVIOs.

### Characterizations

The structure of PI was analyzed with NMR (INOVA‐400MHz, Varian). The light transmittances of the PI and PI/FVIO solutions were measured using a temperature‐controlled UV‐1700 spectrophotometer (SHIMADZU, Japan) at 660 nm, with a heating rate of 0.5 °C/2 min. The cloud‐point temperature was defined as the temperature corresponding to the initial breakpoints in the resultant transmittance versus temperature curve. The crystal phase of PI/FVIOs was verified based on the XRD patterns that were recorded using a powder X‐ray diffractometer (XRD, D/max‐2200/PC, Rigaku, 40 kV, 20 mA, Cu Ka radiation). The particle size and shape of the samples were examined under TEM (FEI Tecnai F30). The hydrodynamic diameter of DOX‐PI/FVIOs or DOX‐FVIOs was determined by dynamic light scattering (DLS, ZEN3600, Malvern Instruments Ltd, UK). The zeta‐potential of the sample was measured as a function of pH at room temperature. The magnetic properties of the samples were characterized using a vibrating sample magnetometer (VSM, Beijing Xinke Technology Development Co., Ltd.). UV–vis absorption was measured using a T6‐1650F spectrophotometer (Persee Instrument Co. Ltd, Beijing, China). L*f*‐EF (30–500 Hz) and M*f*‐EF (220–470 kHz) were operated by different instruments. The L*f*‐EF instrument was programmable alternative AC/DC power (NF, EC750SA/EC1000SA; Japan), and M*f*‐EF instrument was an induction heating system (M5, Xi'an SuperMag Nano‐biotechnology Co. Ltd). The detailed descriptions for instruments regarding EF are provided in Section S5, Supporting Information.

### Cell Culture and In Vitro Studies

Adriamycin‐resistant MCF‐7 (MCF‐7/ADR) cells were purchased from FuHeng Cell Center, Shanghai, China. MDA‐MB‐231 cells were purchased from ATCC. BxPC‐3 cells were obtained from the Cell Bank of the Chinese Academy of Sciences (Shanghai, China). A short tandem repeat DNA profiling method was used to authenticate the cell lines and the results were compared with the reference database.

The cells were incubated in Dulbecco's Modified Eagle Medium (DMEM, Hyclone, USA) (MCF‐7/ADR, 3T3, BxPC‐3) or Roswell Park Memorial Institute 1640 (RPMI‐1640, Hyclone, USA) (MDA‐MB‐231) that contained 10% fetal bovine serum (FBS, Hyclone, USA) and 1% penicillin/streptomycin solution (Gibco) in a humidified atmosphere of 5% CO_2_ at 37 °C.

### Animals Used in the In Vivo Studies

BALB/c nude mice (6–8 weeks, female and male) were purchased from Beijing Charles River Company and they were raised in an Individually Ventilated Cage. All animal experimental protocols were reviewed and approved by the Animal Care and Use Committee of the Institute of Process Engineering, Northwest University, and they complied with all relevant ethical regulations.

### Subcutaneous Tumor Model

For the MCF‐7/ADR model, female BALB/c nude mice were inoculated with MCF‐7/ADR cells (1 × 10^7^ cells per mouse) via subcutaneous injection into the breast fat pad below the abdomen. For the MDA‐MB‐231 and BxPC‐3 models, 1 × 10^7^ cells per mouse were inoculated subcutaneously into the flank of BALB/c nude mice. The mice were randomly divided into different treatment groups. Doxil, DOX‐FVIOs, or DOX‐PI/FVIOs (2.5 mg kg^−1^ DOX per mouse body weight) were injected intravenously (n = 6 or 5 mice per group). The control group received saline injections. All animals received a single injection. Tumor volumes were calculated by the following formula, V = L × W^2^ × 1/2 (V, volume; L, length; W, width of tumor).

### Penetration of PI/FVIO Nanovehicles into MCF‐7/ADR Tumor Spheroids

MCF‐7/ADR breast tumor spheroids were formed in pipet tips containing 3T3 fibroblasts and MCF‐7/ADR breast cancer cells according to the previously described method.^[^ ^58^ ^]^ MCF‐7/ADR tumor spheroids were transferred to a petri dish and they were incubated with PI/FVIOs^FITC^ (10 µg mL^−1^ Fe) for 1–2 h. Then, the MCF‐7/ADR tumor spheroids were washed with PBS and they were then treated with L*f*‐EF (0.03, 0.06, 0.1, 0.3, and 0.5 kHz) or M*f*‐EF (360 kHz) for 10 min. The images were taken using a fluorescence microscope (Nikon TS 2).

### Penetration of DOX‐PI/FVIOs into Subcutaneous MCF‐7/ADR Tumors

BALB/c nude mice bearing MCF‐7/ADR tumors (tumor volume of ≈80 mm^3^) were intravenously injected with DOX‐PI/FVIOs^FITC^ at a dose of 5 mg Fe/kg body weight. After 6 h, the mice were exposed to L*f*‐EF (0.1 kHz) for 10 min. Then, the mice were sacrificed. The tumors were separated and then subsequently prepared for paraffin sectioning. The tumor samples were sectioned into 4 µm‐thick slices along the equatorial plane. Then, the tumor samples were incubated with cy5‐CD31 antibody to label the tumor vessels. After several washes with PBS, the tumor samples were stained with DAPI for 15 min. Finally, the stained sections were scanned and observed under a pathological section scanner (Panoramic MIDI, 3D Histech, Hungary). The 3D structure of the tumor was reconstructed using 3D View software. The penetration and distribution of DOX‐PI/FVIOs^FITC^ in the tumor were analyzed using FIJI (FIJI Is Just Image J).

### Endocytosis of DOX‐PI/FVIOs in MCF‐7/ADR Cells

MCF‐7/ADR cells seeded at a density of 10^4^ cells/well in a 35 mm culture dish were cultured for 24 h. Then, the growth medium was removed and the cells were washed 3 times with PBS. The lysosomes were stained with LysoTracker Deep Red (50 nm, Invitrogen) for 15 min, and the cytomembrane was labeled with DiO (1 µm, Solarbio), according to the manufacturer's instructions, while the nuclei were stained with Hoechst 33 342 (5 µg mL^−1^, Beyotime) for 15 min. The DOX‐PI/FVIOs with a DOX concentration of 12.5 µg mL^−1^ were then added. Live imaging of the cells was performed using a CLSM (Nikon A1 Rsi, Japan) in an environmental chamber to maintain a temperature of 37 °C and an atmosphere of 5% CO_2_. The images were recorded every 5 min for 2 h, and they were then converted into an AVI movie.

### DOX Release

DOX‐PI/FVIOs dispersed in PBS (100 µg mL^−1^ Fe) were placed in the center of a styrofoam‐insulated magnetic induction coil with different *f* (0.1, 220, 260, 320, 360, and 470 kHz) for 10 min. The samples without EF exposure were used as controls. At the designated time intervals, the DOX release process was terminated by switching off the EF. Subsequently, 1 mL supernatant was extracted and then investigated by UV–vis spectroscopy to determine the concentration of the released DOX. The temperature profile of the sample was recorded using a fiber thermocouple.

The DOX release induced by the heat generated at the surface of DOX‐PI/FVIOs (50 µg mL^−1^ Fe) under EF with different *f* (0.1, 220, 260, 320, 360, and 470 kHz) was observed under UV light (365 nm). Meanwhile, an IR thermal imaging camera (Fotric 225) was used to monitor the temperature of the solution. Samples were illuminated under UV light (365 nm)

### Intracellular DOX Release

For the intracellular DOX release in M*f*‐EF (360 kHz) study, 1 × 10^5^ MCF‐7/ADR cells/well were cultured in glass‐bottomed dishes for 24 h, and then DOX‐PI/FVIOs or DOX‐FVIOs (12.5 µg mL^−1^ DOX) were added, before subjecting to incubation for another 12 h. The cells were washed with PBS and the nuclei were stained with Hoechst 33 342. After that, the cells were exposed to M*f*‐EF (360 kHz, 300 Oe) for 10 min. The images were recorded using CLSM (Nikon A1 Rsi).

After staining with Hoechst 33 342, the cells were exposed to M*f*‐EF (360 kHz), and images were obtained using CLSM (Nikon A1 Rsi) every 1 min. The images were later converted into an AVI/mp4 movie. The percentage of DOX fluorescence in nuclei was subsequently analyzed using a HistoQuest system (TissueGnostics GmbH), following the manufacturer's recommendations.

### Flow Cytometry Measurement

Cells were seeded into a 6‐well plate with a density of 1 × 10^5^ per well, and the cells were incubated for 24 h before subjecting them to the treatment with either Doxil or DOX‐PI/FVIOs (10 µg mL^−1^ DOX) under 10 min M*f*‐EF. After that, the cells were incubated in a fresh medium for either 4 or 8 h. The cells were then trypsinized, washed three times with cold PBS, and re‐suspended in 4 mL PBS. The cells were immediately analyzed using flow cytometry (CyFlow Space). Files were collected for 10 000 gated events, and three independent duplicated experiments were conducted.

### In Vitro Cell Cytotoxicity Assay

The cytotoxicity of DOX‐PI/FVIOs was determined using a cell counting kit‐8 assay (CCK‐8, Goyoo Bio Co., Ltd.) based on a modified manufacturer's protocol. Briefly, MCF‐7/ADR cells were seeded in a 6‐well plate at a density of 10^5^ cells per well and they were incubated for 24 h. Subsequently, the treatments, that is, Doxil, DOX‐FVIOs, or DOX‐PI/FVIOs, of certain DOX concentrations, were added to the well. Cells were incubated for 24 h and they were then detached from the culture wells. For DOX‐FVIOs and DOX‐PI/FVIOs groups, the cell suspensions were placed into Eppendorf tubes, and then they were exposed to a M*f* EF (300 Oe, 360 kHz) for 10 min. The cells for all treatment groups were then reseeded in a 96‐well plate containing fresh medium, and it was incubated for another 24 h before performing the CCK‐8 assay. The absorbance was measured at 450 nm using a Varioskan Flash (Thermo Fisher Scientific, USA). Cell viability was determined as a percentage of untreated control cells, and the IC_50_ values were determined by the nonlinear regression analyses obtained from Graphpad Prism 8 software.

### Efficiency of DOX Delivery to the Nucleus in MCF‐7/ADR Subcutaneous Tumors

To investigate the DOX delivery efficiency of various treatments, nude mice bearing MCF‐7/ADR tumors (≈50 mm^3^) were divided into seven groups: 1) Doxil, 2) DOX‐PI/FVIOs, 3) DOX‐PI/FVIOs + L*f*‐EF (0.1 kHz, 10 min), 4) DOX‐PI/FVIOs + M*f*‐EF (360 kHz, 300 Oe, 10 min), 5) DOX‐FVIOs + L*f*‐EF (0.1 kHz, 10 min) & M*f*‐EF (360 kHz, 300 Oe, 10 min), 6) DOX‐PI/FVIOs + M*f*‐EF (360 kHz, 300 Oe, 10 min) & L*f*‐EF (0.1 kHz, 10 min), and 7) DOX‐PI/FVIOs + L*f*‐EF (0.1 kHz, 10 min) & M*f*‐EF (360 kHz, 300 Oe, 10 min). The mice were intravenously injected with the samples at a similar dose of 2.5 mg DOX/kg body weight. The time interval of programmable EF was 2 h (Figure S16, Supporting Information). Next, the mice were euthanized and the tumor was separated for paraffin sectioning. The sections were incubated with DAPI to label the nuclei of the tumor cells. The sections were inspected under a PerkinElmer Operetta CLS. The percentage of DOX fluorescence in the nucleus was analyzed with the HistoQuest system (Tissue Gnostics) according to the manufacturer's recommendations.

### Subcutaneous MCF‐7/ADR Tumor Model

The subcutaneous MCF‐7/ADR tumor model was established according to the method described above. After two weeks, the mice were randomly assigned to one of the eight groups (n = 6 per group), and they were intravenously administered with 100 µL of different formulations at an equal DOX dose of 2.5 mg kg^−1^ body weight, respectively. The groups were: 1) Doxil, 2) DOX‐PI/FVIOs, 3) DOX‐PI/FVIOs + L*f*‐EF (0.1 kHz, 10 min), 4) DOX‐PI/FVIOs + M*f*‐EF (360 kHz, 300 Oe, 10 min), 5) DOX‐FVIOs + L*f*‐EF (0.1 kHz, 10 min) & M*f*‐EF (360 kHz, 300 Oe, 10 min), 6) DOX‐PI/FVIOs + M*f*‐EF (360 kHz, 300 Oe, 10 min) & L*f*‐EF (0.1 kHz, 10 min), and 7) DOX‐PI/FVIOs + L*f*‐EF (0.1 kHz, 10 min) & M*f*‐EF (360 kHz, 300 Oe, 10 min). Saline treatment was set as the control group. The treatment consisted of a single injection. An IR thermal imaging camera was used to observe the temperature of the tumor. The body weights of the mice were monitored. The mice were eventually sacrificed at 39 d post‐inoculation, and the tumors were excised, weighed, and photographed.

### Subcutaneous MDA‐MB‐231 Breast Tumor Model

6–8 weeks old female BALB/c nude mice were inoculated with MDA‐MB‐231 cells (1 × 10^7^ cells per mouse) via subcutaneous injection into their left flank. When the tumor volume reached ≈80 mm^3^, the mice were randomly assigned into one of five groups (n = 5 mice per group), and they were intravenously administered with 100 µL saline or different DOX formulations at a 2.5 mg kg^−1^ DOX‐equivalent dose. The five groups were as follows: 1) Saline, 2) Doxil, 3) DOX‐FVIOs + L*f*‐EF (0.1 kHz, 10 min) & M*f*‐EF (360 kHz, 300 Oe, 10 min), 4) DOX‐PI/FVIOs + M*f*‐EF (360 kHz, 300 Oe, 10 min) & L*f*‐EF (0.1 kHz, 10 min), and 5) DOX‐PI/FVIOs + L*f*‐EF (0.1 kHz, 10 min) & M*f*‐EF (360 kHz, 300 Oe, 10 min). The length and width of the tumors, as well as the bodyweight of the mice, were monitored.

### Subcutaneous BxPC‐3 Pancreatic Tumor Model

In brief, BxPC‐3 cells were harvested and resuspended at a density of 2 × 10^8^ cells mL^−1^ in PBS buffer. Then, male BALB/c nude mice were inoculated with BxPC‐3 cells via subcutaneous injection into their right flank. The assignment of mice to the different groups and treatment procedures for the pancreatic tumor model were similar to those in the MDA‐MB‐231 tumor model. The experiment was terminated 95 d after the cell inoculation.

### Toxicity Study

Toxicity experiments were carried out on SD rats (male and female, 180–200 g). The rats were injected with DOX‐PI/FVIOs (5 mg Fe/kg body weight) or an equal volume of saline (n = 3/group) through the tail vein. After 1 d or 14 d, the rats were euthanized. Blood was collected for routine blood cell counts and serum was collected to analyze biochemical indicators of liver function. Indicators of hepatocyte damage (ALT: alanine aminotransferase, AST: aspartate aminotransferase, ALP: alkaline phosphatase), indicators of hepatocyte protein anabolism function (TP: total protein and ALB: albumin), and indicators of liver excretion, secretion, and detoxification (TBIL: total bilirubin, DBIL: direct bilirubin) were all measured using an automatic biochemistry analyzer (Chemray 240, Rayto Life and Analytical Sciences Co. Ltd.). The brain, heart, kidney, liver, lung, and spleen were also collected after 1 d or 30 d for H&E staining. The sections were observed under a pathological section scanner.

### Statistical Analysis

The in vitro experiments were performed three times independently. All animal studies were performed after randomization. Student's *t*‐test, one‐way, and two‐way ANOVA were used for multiple comparisons as described in the figure legends. *P* < 0.05 was regarded as a significant difference (*); *P < 0.05; **P < 0.01; ***P < 0.001; ****P < 0.0001. Differences in the animal survival were calculated using the Kaplan–Meier method and the *P* value was obtained by the log‐rank (Mantel‐Cox) test.

## Conflict of Interest

The authors declare no conflict of interest.

## Supporting information

Supporting Information

Supporting Movie 1

Supporting Movie 2

Supporting Movie 3

Supporting Movie 4

Supporting Movie 5

Supporting Movie 6

## Data Availability

Research data are not shared.
