# Supplementary material for: Electromagnetic Field‐Programmed Magnetic Vortex Nanodelivery System for Efficacious Cancer Therapy
Source: Adv Sci (Weinh). 2021 Jul 18;8(18):2100950. doi: 10.1002/advs.202100950 (PMC8456207; doi:10.1002/advs.202100950)
Supplement: Supplementary file 1 — Supporting Information [file ADVS-8-2100950-s004.pdf]

WILEY-VCH

© 2021 Wiley-VCH GmbH

ADVANCED SCIENCE
Open Access

Supporting Information

for *Adv. Sci.*, DOI: 10.1002/advs.202100950

Electromagnetic Field-Programmed Magnetic Vortex Nanodelivery System for Efficacious Cancer Therapy

Xiaoli Liu, Yifan Zhang, Yu Guo, Wangbo Jiao, Xiao Gao, Wee Siang Vincent Lee, Yanyun Wang, Xia Deng, Yuan He, Ju Jiao, Ce Zhang, Guoqing Hu, Xing-Jie Liang and Haiming Fan**

Supporting Information

Electromagnetic Field-Programmed Magnetic Vortex Nanodelivery System for Efficacious Cancer Therapy

Xiaoli Liu,⊥ Yifan Zhang,⊥ Yu Guo, Wangbo Jiao, Xiao Gao, Wee Siang Vincent Lee, Yanyun Wang, Xia Deng, Yuan He, Ju Jiao, Ce Zhang, Guoqing Hu, Xing-Jie Liang and Haiming Fan**

Prof. X. Liu, X. Gao, Prof. H. M. Fan
Key Laboratory of Resource Biology and Biotechnology in Western China, Ministry of Education; School of Medicine, Northwest University, Xi'an, Shaanxi 710069, China
E-mail: fanhm@nwu.edu.cn;

Prof. X. Liu, Prof. X.-J. Liang
CAS Key Laboratory for Biomedical Effects of Nanomaterials and Nanosafety, CAS Center for Excellence in Nanoscience, National Center for Nanoscience and Technology of China, No. 11, First North Road, Zhongguancun, Beijing 100190, China
University of Chinese Academy of Sciences, Beijing 100049, China
E-mail: liangxj@nanoctr.cn;

Y. Zhang, W. Jiao, Y. Wang, Prof. Y. He, Prof. H. Fan
Key Laboratory of Synthetic and Natural Functional Molecule Chemistry of the Ministry of Education, College of Chemistry and Materials Science, Northwest University, Xi'an 710127, China

Prof. Y. Guo, Prof. G. Hu
Department of Engineering Mechanics, Zhejiang University, Hangzhou 310027, China

Dr. W. S. V. Lee
Department of Materials Science and Engineering, National University of Singapore, 117573 Singapore

X. Deng
School of Life Sciences and Electron Microscopy Center of Lanzhou University, Lanzhou University, Lanzhou 730000, China

Dr. J. Jiao
Department of Nuclear Medicine, The Third Affiliated Hospital of Sun Yat-sen University, 600 Tianhe Road, Guangzhou, Guangdong 510630, China

Prof. C. Zhang
State Key Laboratory of Cultivation Base for Photoelectric Technology and Functional Materials, Laboratory of Optoelectronic Technology of Shaanxi Province, National Center for International Research of Photoelectric Technology & Nanofunctional Materials and Application, Institute of Photonics and Photon-Technology, Northwest University, Xuefu Street No. 1, Xi'an 710127, China

Section S1. Calculating the diffusion coefficient of PI/FVIOs^{FITC}

Labeling of PI/FVIOs with FITC. PI/FVIOs were dispersed in 1 mL DI water, and the pH was adjusted to 8.0 using sodium bicarbonate. FITC-NHS dissolved in PBS (3 mg/mL) was added to the above prepared solution. After shaking for 3 h, the solution was centrifuged at 6000 rpm for 10 min, and it was then washed with DI water several times until no fluorescence was detected in the supernatant. The as-produced PI/FVIOs^{FITC} were then re-dispersed in PBS buffer.

Calculating the diffusion coefficient of PI/FVIOs^{FITC}. PI/FVIOs^{FITC} diffused from the outside of the tumor spheroid to the inside, which results in a lower grey value near the geometric centre of the tumor spheroid in the grayscale image. The efficient diffusion of PI/FVIOs^{FITC} throughout the tumor spheroid can be evaluated using the following factors: (1) The grey value of pixels increases near the centre of the tumor spheroid, and (2) there is a reduced distance between the pixel with the minimum grey value and the centre of the tumor spheroid. The parameter *P* is defined to analyze the distribution of PI/FVIOs^{FITC} in a tumor spheroid:

$$P = \frac{\sum_i D_i * R_i}{N}$$

where, D_i is the grey value of the point pixel in an image, R_i is the Euclidean distance from the pixel to the geometric centre of the tumor spheroid, and *N* is the number of pixels in the tumor spheroid.

After normalizing both D_i and R_i, the modified formula can be presented as follows:

$$P = \frac{\sum_i \frac{D_i}{255} * \frac{R_i}{500}}{N}$$

where, the value $\frac{D_i}{255}$ is the grey value in the range [0,1], and the value $\frac{R_i}{500}$ is the distance in the range [0,1].

The parameter r is proposed to evaluate the diffusion capacity of PI/FVIOs^{FITC} in the tumor spheroid under L*f*-EF, and it can be determined using the equation presented as follows:

$$r = \frac{P_{after}}{P_{before}}$$

where, P_{before} and P_{after} represent the distribution of PI/FVIOs^{FITC} in the tumor spheroid before and after L*f*-EF treatment, respectively. Theoretically, the r value is expected to be less than 1, and the smaller the r value, the higher the diffusion capacity of PI/FVIOs^{FITC}.

Section S2. Numerical modeling of the penetration by PI/FVIOs

A three-dimensional channel filled with ellipsoidal cells was numerically modeled (as shown in Figure S5a). Five FVIOs are generated at the top of the vertical channel. A constant force F_0 (accounting for the chemical potential) is exerted on each FVIOs. This drives the FVIOs to move downwards through the voids in the matrix of the positionally fixed cells. Periodic boundary conditions were used in the two horizontal directions (x and z directions). Due to the combined effects of the alternating magnetic force and the interaction with surrounding substances, a random force F_R was applied to each nanomaterial. The direction of F_R changed randomly at the same frequency with that of the EF. The translational motion of individual FVIO is governed by Newton's second law of motion,

$$m_i \frac{\mathrm{d}\mathrm{v}_i}{\mathrm{d}t} = F_0 + F_R + F_C \ , \qquad (1)$$

in which v_i is the translational velocity vector of the nanomaterial i with mass m_i. When the nanomaterials hit the cells, the contact force F_C between them can be calculated as:

$$F_C = \begin{cases} k\delta_n e_n, & \delta_n \leq 0 \\ F_\sigma e_n, & 0 < \delta_n \leq \delta_\sigma \\ 0, & \delta_\sigma < \delta_n \end{cases} . \qquad (2)$$

The distance between a FVIO and a cell, δ_n, was determined to calculate the repulsive contact force, $k\delta_n$, for $\delta_n \leq 0$, and the cohesive contact force, F_σ, for $0 < \delta_n < \delta_\sigma$. The symbol k represents the contact stiffness between a FVIO and a cell, and e_n is the unit vector of contact normal direction. The contact force vanishes when the distance δ_n is greater than a critical value δ_σ, which indicates the detachment of the nanomaterial from the cell. In the present simulations, the magnitudes of F_R and F_C were set to twice that of the potential force F_0.

The penetrations by the FVIOs through the cell-filled channel at various EF frequencies were simulated. The time evolution of the average displacements of nanomaterials is shown in Figure S5b, in which the average displacement D was scaled by the nanomaterial diameter d, and the time t was scaled by the characteristic time t_0, *i.e.*, the time required for a FVIO to

travel by a channel distance subjected to a potential force F_0 (with no contact force F_C and no random force F_R). It is evident that FVIOs could barely move in a static magnetic field and they moved by a small displacement at low EF frequencies, *i.e.*, f = 0.03 and 0.06 kHz. The transportation of FVIOs was significantly improved by increasing the EF frequencies to 0.1 and 0.3 kHz. However, as the EF frequency was raised to 0.5 kHz, the movement of FVIOs was significantly reduced to the level of that in the static magnetic field. The difference in the transportation of nanomaterials at various EF frequencies could also be observed in the snapshots at time t/t_0 = 1400 (as shown in Figure S5c). The present simulation results are in good agreement with the experimental observation. Thus, the random forces induced by the EF can disturb the motion of FVIOs, which increases the thermal dynamics and reduces the chances for FVIOs to be trapped on the rough and sticky surfaces of the cells. A very low-frequency EF is close to a static magnetic field and too high a frequency can lead to the leveling-out of the disturbance by averaging over a short period of time. As a result, the best performance is achieved at an optimal EF frequency.

Section S3. Analyzing the distribution of PI/FVIOs^{FITC} fluorescence signal in solid tumor

L*f*-EF displays a gradient field distribution. To investigate the tumor penetration ability of PI/FVIOs under the influence of L*f*-EF, we sliced the tumors along three mutually perpendicular axes (x, y, and z axes), and then we observed the diffusion and distribution of PI/FVIOs^{FITC} in the tumor using a fluorescence microscope. The direction parallel to the central axis of the coil was denoted as the z-axis direction of the tumor (x-y plane); the other two directions were the x-axis (y-z plane) and the y-axis (x-z plane). The fluorescence intensity of PI/FVIOs^{FITC} after L*f*-EF actuation at the tumor x-y plane remained to be over 48%, and it was also kept at about 30% at the y-z plane and x-z plane. In contrast, the fluorescence intensity at any plane of the control group was only around 12-17%. The results show that after L*f*-EF exposure, the diffusion of PI/FVIOs^{FITC} into the tumor was significantly enhanced along the z-axis of the tumor. In the other two directions, PI/FVIOs^{FITC} showed a limited diffusion enhancement as compared to that in the z-axis direction. However, it is worth noting that their diffusion abilities (along x and y axes) were still higher than those of the group without L*f*-EF exposure. Such a result is mainly due to the larger gradient of L*f*-EF along the z-axis direction (x-y plane).

Section S4. Measurement of SAR values

Quantitation of heat generation by the samples was performed using an induction heating system (M5, Xi'an SuperMag Nano-biotechnology Co. Ltd). 1 mL sample was exposed to M*f*-EF (300 Oe) at various magnetic field frequencies. The temperature rise profiles were then recorded using an optical fiber thermocouple. The SAR was determined using the following equation:

$$\mathrm{SAR} = \mathrm{C}\frac{\Delta T}{\Delta t}\frac{1}{m_{Fe}}$$

where, C is the specific heat of the medium (C_{water} = 4.18 J g^{-1}°C^{-1}), ΔT/Δt is the initial slope of the time-dependent temperature curve, and m_{Fe} is the weight fraction of Fe in the medium. The concentrations of Fe in the samples were determined using ICP-MS (Agilent 7900).

Section S5. The descriptions of the EF-generating instruments

L*f*-EF (30-500 Hz) and M*f*-EF (220-470 kHz) were operated using different instruments.

The instrument used to produce L*f*-EF (30-500 Hz) (Section S5-1) is described as follows: A Cu coil with 300 turns of wires was used to generate the L*f*-EF, and it was driven by a programmable alternative AC/DC power (NF, EC750SA/EC1000SA; Japan). The power supply was operated in AC mode as the voltage source and output was a sinusoidal alternating voltage (30-500 Hz, 10 V).

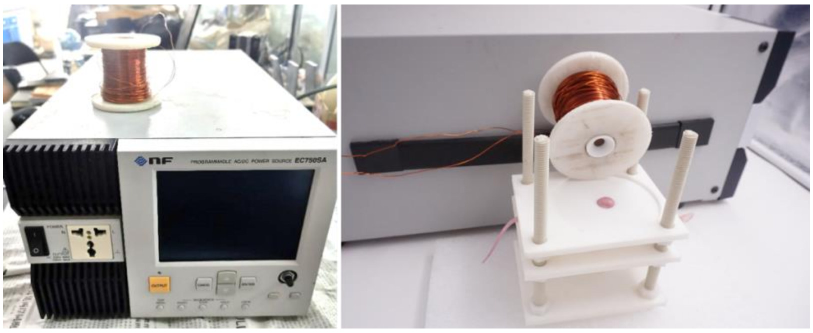

Section S5-1. Digital photograph of the L*f*-EF instrument.

The instrument used to produce M*f*-EF (220-470 kHz) (Section S5-2) is described as follows: M*f*-EF was generated by an induction heating system (M5, Xi'an SuperMag Nano-biotechnology Co. Ltd). The frequency was modulated by changing the coils with the different diameter and turns.

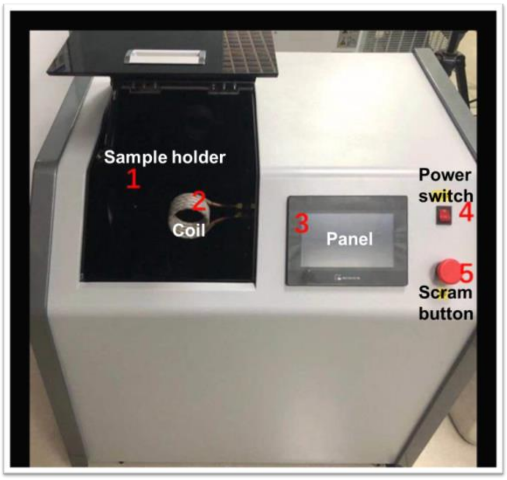

Section S5-2. Digital photographs of the M*f*-EF instrument.

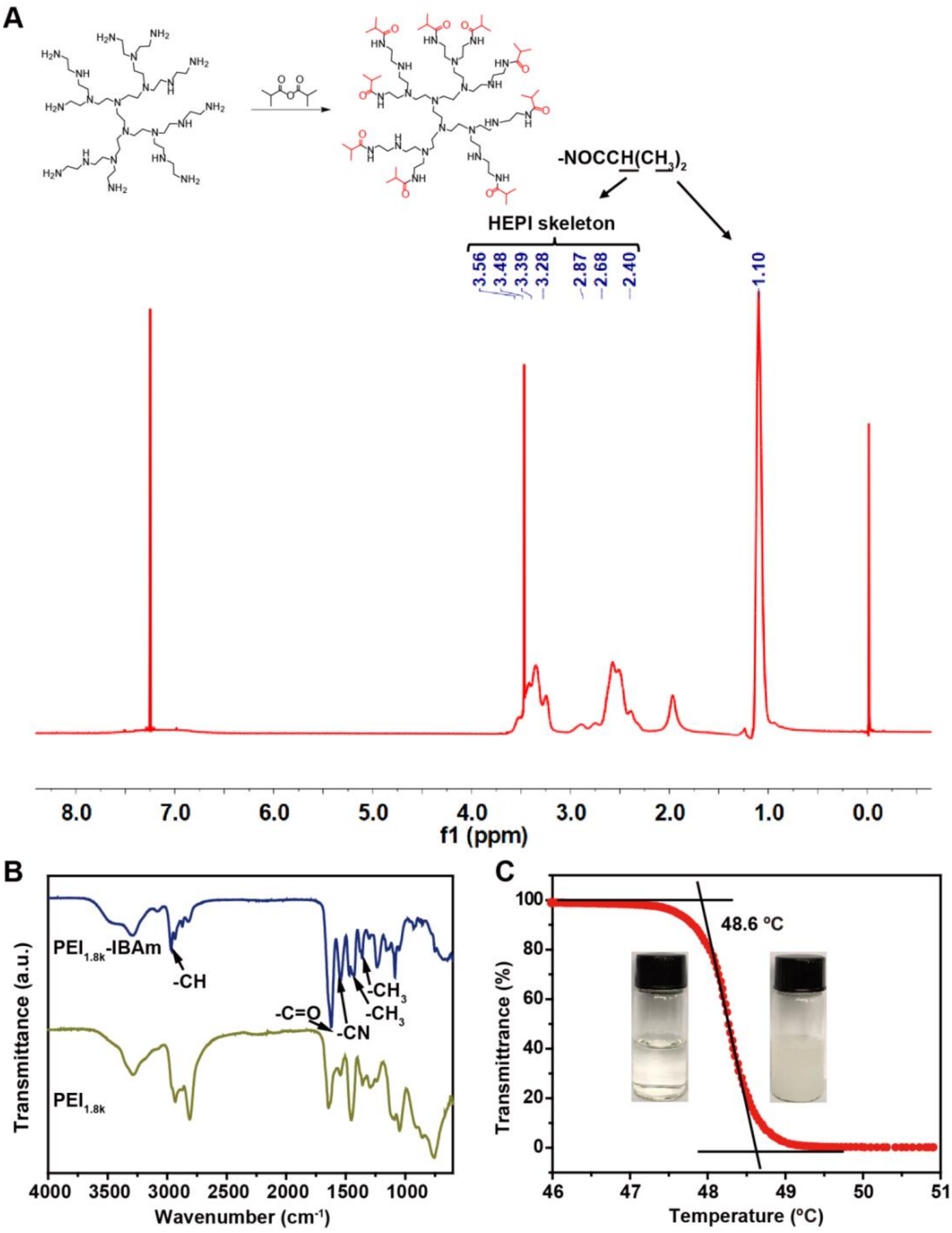

Figure S1. Characterization of thermoresponsive PEI$_{1.8k}$-IBAm (PI). (**A**) ^{1}H-NMR spectra of PI. Experiments were repeated three times. (**B**) FTIR spectrums of PEI$_{1.8k}$ and PI. Experiments were repeated three times. (**C**) Light transmittance of PI aqueous solution at different temperatures. Experiments were repeated three times. Inset: Photographs of PI below LCST, *i.e.*, room temperature, (left) and PI above LCST, *i.e.*, 52°C, (right).

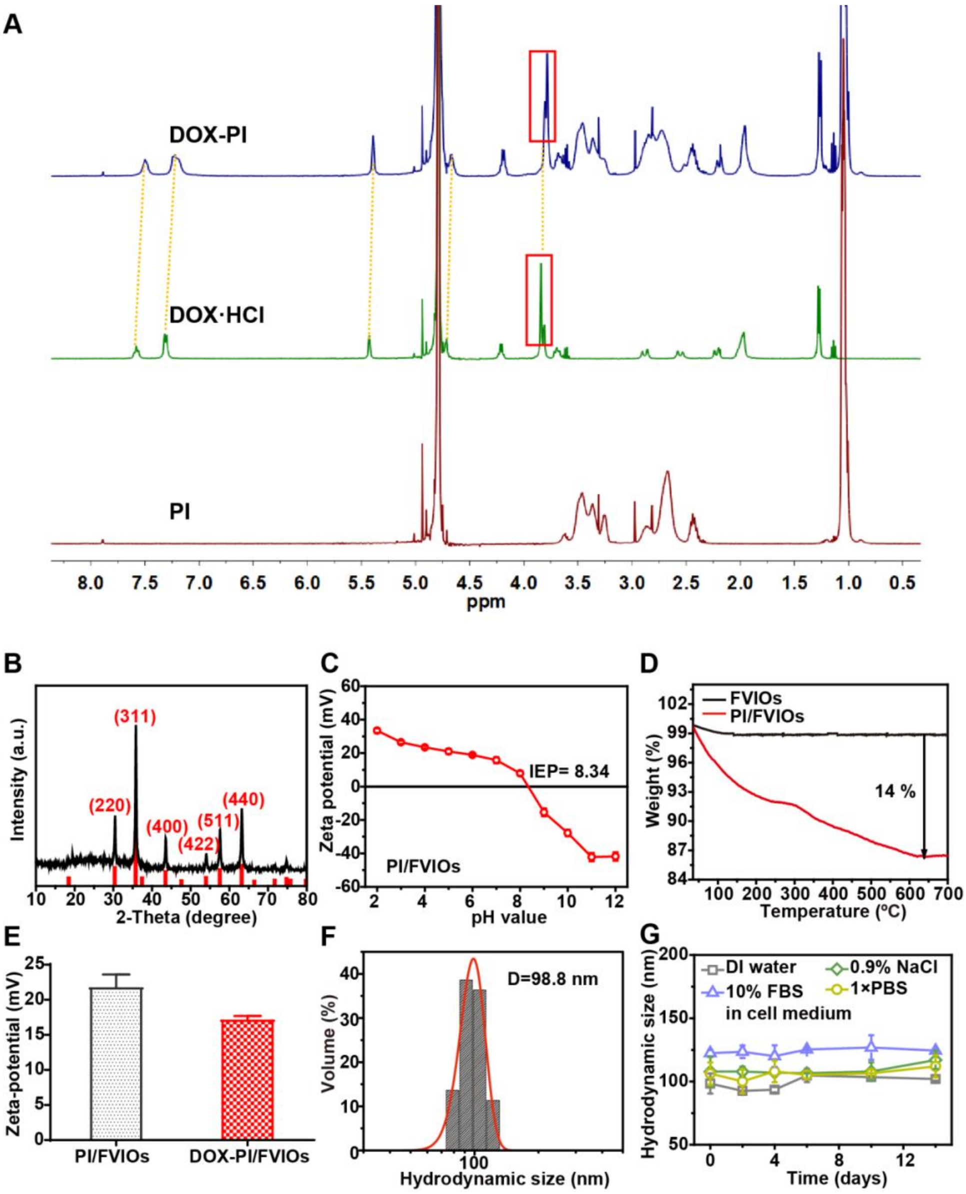

Figure S2. Characterization of PI/FVIOs and DOX-PI/FVIOs. (**A**) ^{1}H-NMR spectra of PI, DOX·HCl, and DOX-PI in D_2O, respectively. Experiments were repeated three times. The characteristic spectrum of DOX·HCl exhibited an upfield shift in the spectrum of the complex (DOX-PI), and its characteristic peaks are of slightly broadened, which may be taken as an evidence for participation of the DOX in the interaction with the PI. (**B**) XRD pattern of PI/FVIO nanovehicles. All diffraction peaks could be exclusively indexed to those of cubic spinel Fe_3O_4 (JCPDS no. 19-0629), without any detected impurities. (**C**) Zeta-potentials of PI/FVIOs at different pH values. PI/FVIO nanovehicles possessed a positive iso-electric point (IEP) of 8.34. (**D**) Thermogravimetric analysis (TGA) of FVIOs and PI/FVIOs. Experiments were repeated three times. The PI/FVIO nanovehicles were composed with 14 wt% PI on the surface of the magnetic core. (**E**) Zeta-potentials of PI/FVIOs (black) and DOX-PI/FVIOs (red). Experiments were repeated three times. (**F**) The hydrodynamic diameter of DOX-

PI/FVIOs in aqueous solution as characterized by dynamic light scattering (DLS). Experiments were repeated three times. The average hydrodynamic diameter of DOX-PI/FVIOs determined by DLS was 98.8 nm. (**G**) Hydrodynamic diameter of DOX-PI/FVIOs measured as a function of incubation time in distilled (DI) water, 1×PBS pH 7.4, 0.9% NaCl, and cell medium containing 10% fetal bovine serum (FBS).

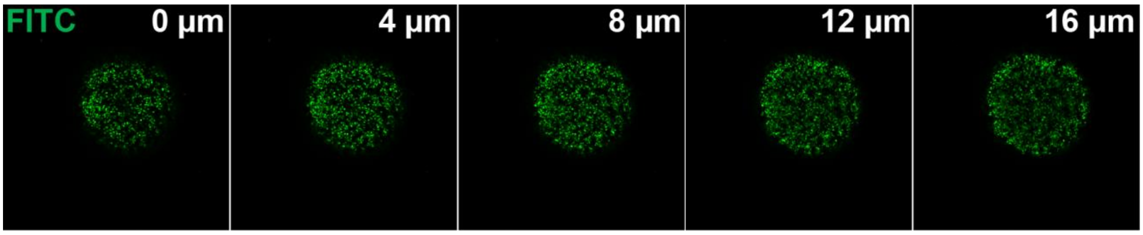

Figure S3. CLSM observations of the penetration by PI/FVIOs. Multilayer CLSM observations of the penetration into MCF-7/ADR breast tumor spheroids by PI/FVIOs^{FITC} *in vitro* under L*f*-EF at 0.1 kHz.

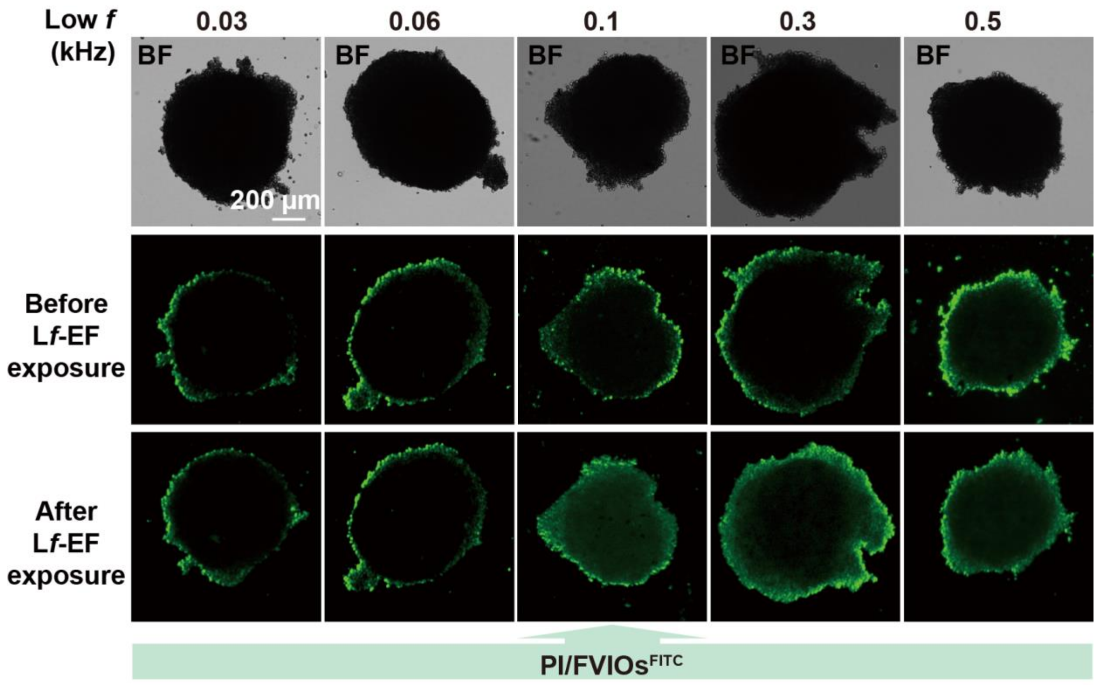

Figure S4. Fluorescence microscopy observations of the penetration by PI/FVIOs. Fluorescence microscopy observations of the penetration into MCF-7/ADR breast tumor spheroids by PI/FVIOs^{FITC} *in vitro* under L*f*-EF at different frequencies.

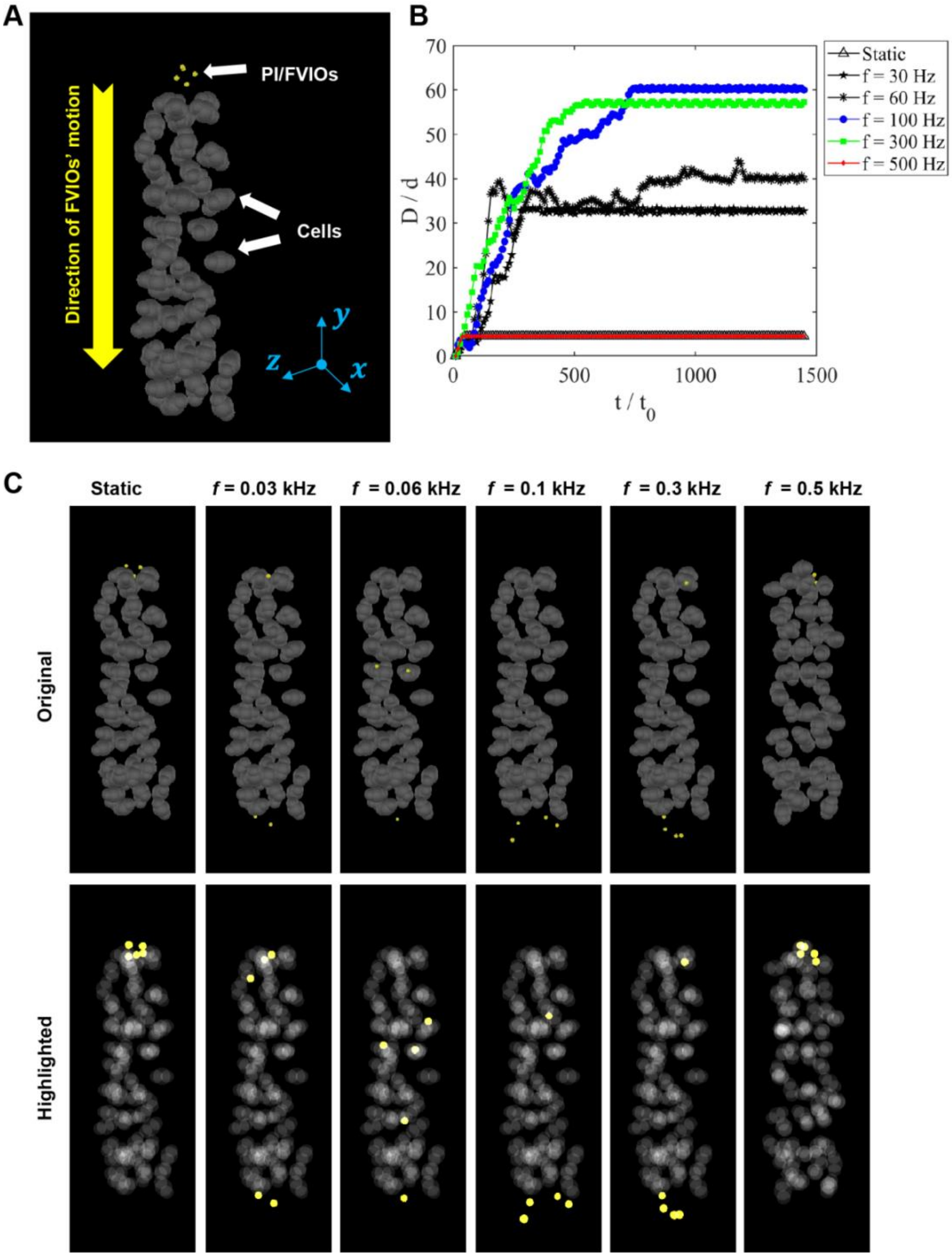

Figure S5. Numerical modeling of the penetration by PI/FVIOs. (**A**) Computational set-up of the tissue penetration by PI/FVIOs. (**B**) Time evolution of the displacements of PI/FVIOs at different EF frequencies. (**C**) Snapshots of PI/FVIOs at time t/t_0 = 1400 for various EF frequencies. The top row shows the original images and the lower row shows the corresponding images in which the PI/FVIOs were enlarged twice and the cells were made translucent to achieve clearer visualization.

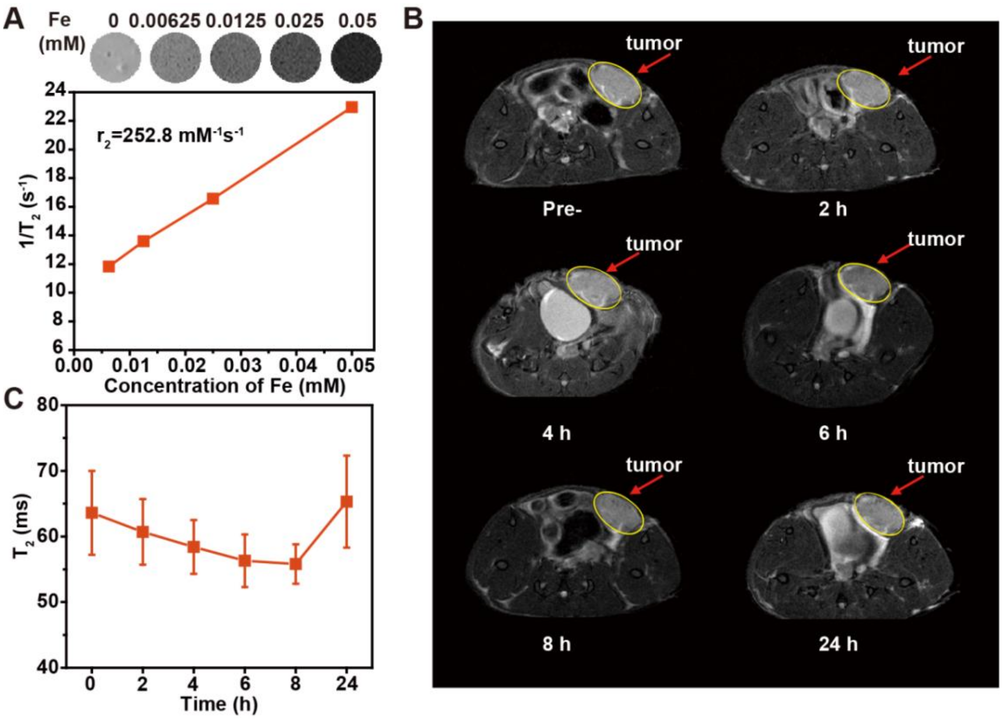

Figure S6. *In vitro* and *in vivo* T_2-weighted magnetic resonance (MR) imaging. (**A**) MR relaxivity measurements of PI/FVIOs aqueous solutions were carried out using a 7 T MRI scanner (Bruker BioSpec, Germany). (**B**) *In vivo* T_2-weighted MR images of subcutaneous breast tumor. MCF-7/ADR tumor-bearing BALB/c nude mice were intravenously injected with DOX-PI/FVIOs (5 mg Fe/kg body weight). *In vivo* MR imaging was performed at various time intervals (pre-injection, and 2 h, 4 h, 6 h, 8 h, and 24 h post-injection) using a 7.0 T small-animal MRI instrument (BioSpec 70/20 USR, Bruker, Germany). When the tumor volume reached a size of ~80 mm^3, the mice were anesthetized with a 2% isoflurane-oxygen mixture in an isoflurane induction chamber. MR images were acquired using a T_2_Turbo RARE sequence with the following parameters: TR=3000 ms, TE=40 ms, field of view=30×30 mm^2, flip angle=90º, matrix size=256×256, and NEX=4. (**C**) T_2 values of the tumor area.

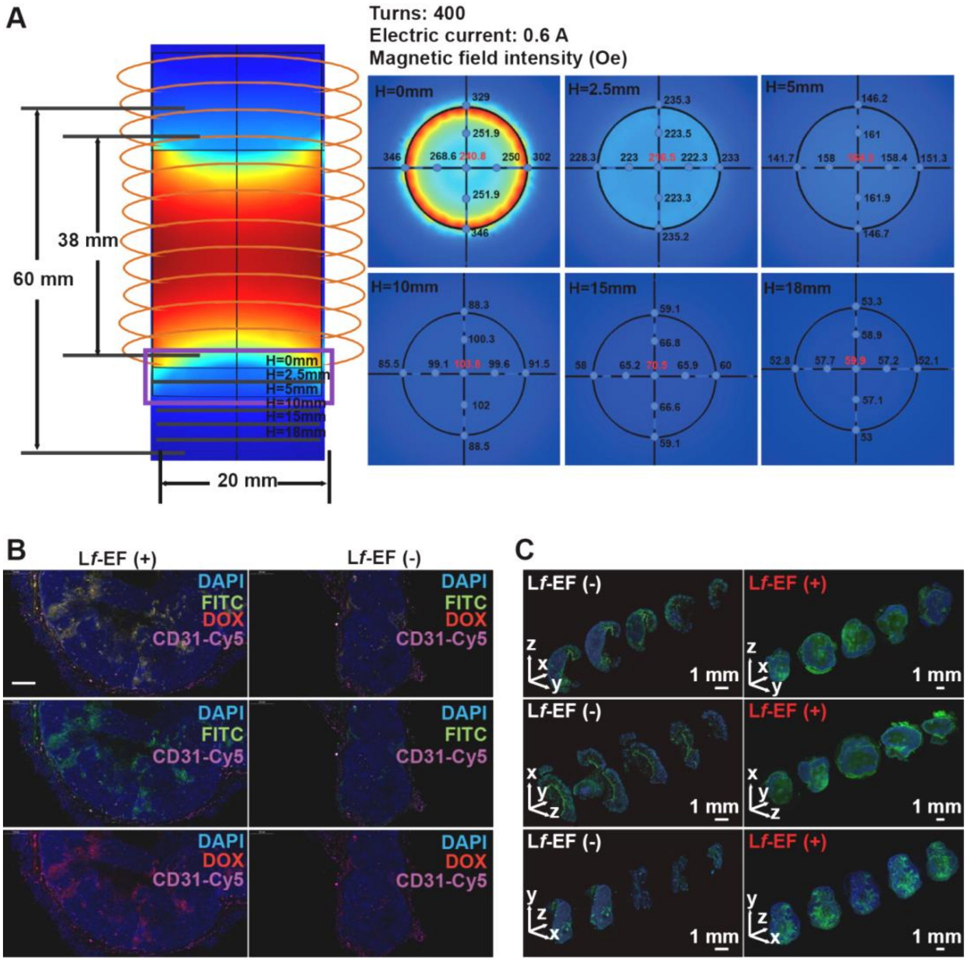

Figure S7. The magnetic field intensity distribution of L*f*-EF and fluorescence images of tumors. (**A**) The magnetic field intensity distribution was simulated by COMSOL using a finite element model (left), and the magnetic field intensity distribution was measured with a Gaussmeter (right). (B) Representative fluorescence images of tumors showing the spreading of DOX-PI/FVIOs from the blood vessels into the tumor tissue after L*f*-EF exposure. Scale bar = 500 µm. (C) Confocal microscopy images of different depths along the three different axes in the tumor tissue. Paraffin-embedded tumor sections were cut into 80 slices in different planes (x, y, z) with a thickness of 4 µm. The images show that the penetration depth of PI/FVIO^{FITC} nanovehicles dramatically increased under L*f*-EF actuation.

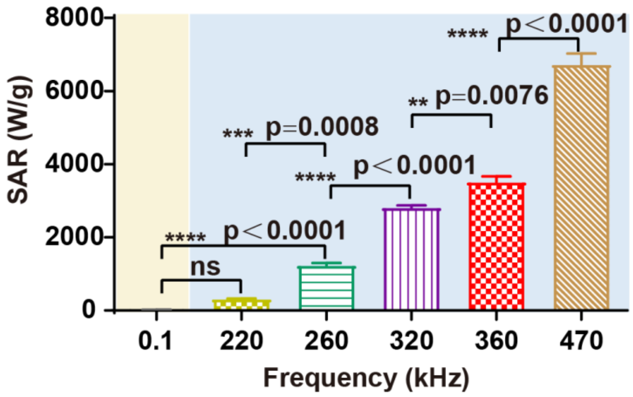

Figure S8. SAR values of DOX-PI/FVIOs. SAR values of DOX-PI/FVIOs (100 μg/mL Fe) under M*f*-EF at 220, 260, 320, 360, and 470 kHz, and L*f*-EF at 0.1 kHz. To determine the differences among the groups, statistical analysis was conducted *via* one-way ANOVA with Tukey's multiple comparison test. p values < 0.05 were considered as statistically significant. The range of p values was indicated by the number of asterisks (*), *i.e.*, *0.01 < p < 0.05; **0.001 < p < 0.01; ***0.01 < p < 0.001; ****0.001 < p < 0.0001. ns, not significant (p > 0.05).

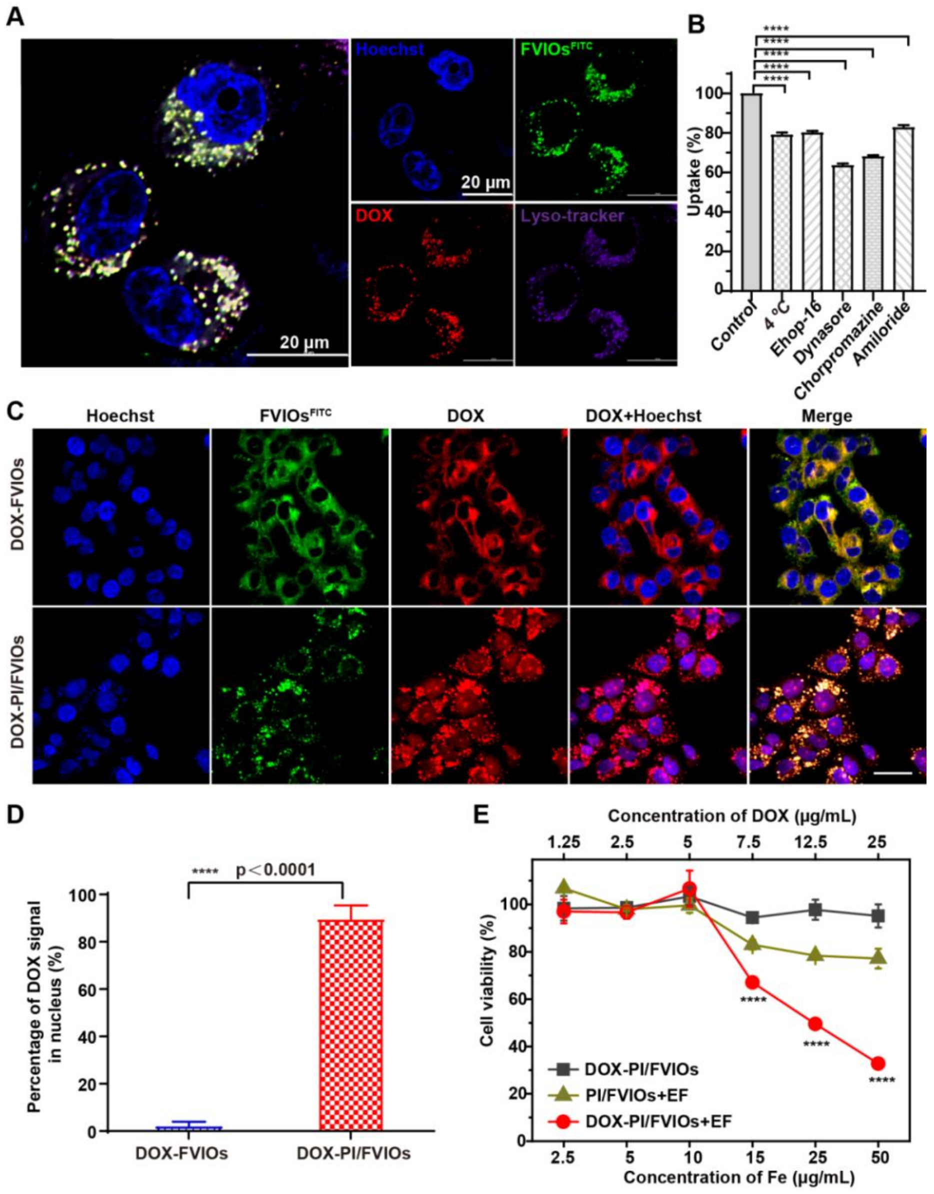

Figure S9. Endocytosis, intracellular DOX release, and cell cytotoxicity assay for MCF-7/ADR cells. (**A**) CLSM images of MCF-7/ADR cells treated with DOX-PI/FVIOs^{FITC} (12.5 μg/mL DOX) for 2 h. (**B**) Endocytosis mechanism studies in MCF-7/ADR cells. Cells were pretreated at 4 ℃ or with ehop-16, dynasore, chlorpromazine, and amiloride for 1h. Fluorescence intensity in the non-treated cells was used as control. Results were represented by mean ± SD, n=3. ****p < 0.0001; statistical analysis was conducted *via* a two-tailed unpaired Student's t test. (**C**) The intracellular DOX distributions after 12 min M*f*-EF exposure. Upper panels: CLSM images of MCF-7/ADR cells treated with DOX-FVIOs or DOX-PI/FVIOs after stimuli with 360 kHz M*f*-EF for 10 min. The nuclei were stained with Hoechst. Scale bar, 50 μm. (**D**) The percentage of DOX signal in the nucleus. Statistical analysis was conducted *via* a two-tailed unpaired Student's t test. (**E**) Cell viability of MCF-7/ADR cells after different treatments.

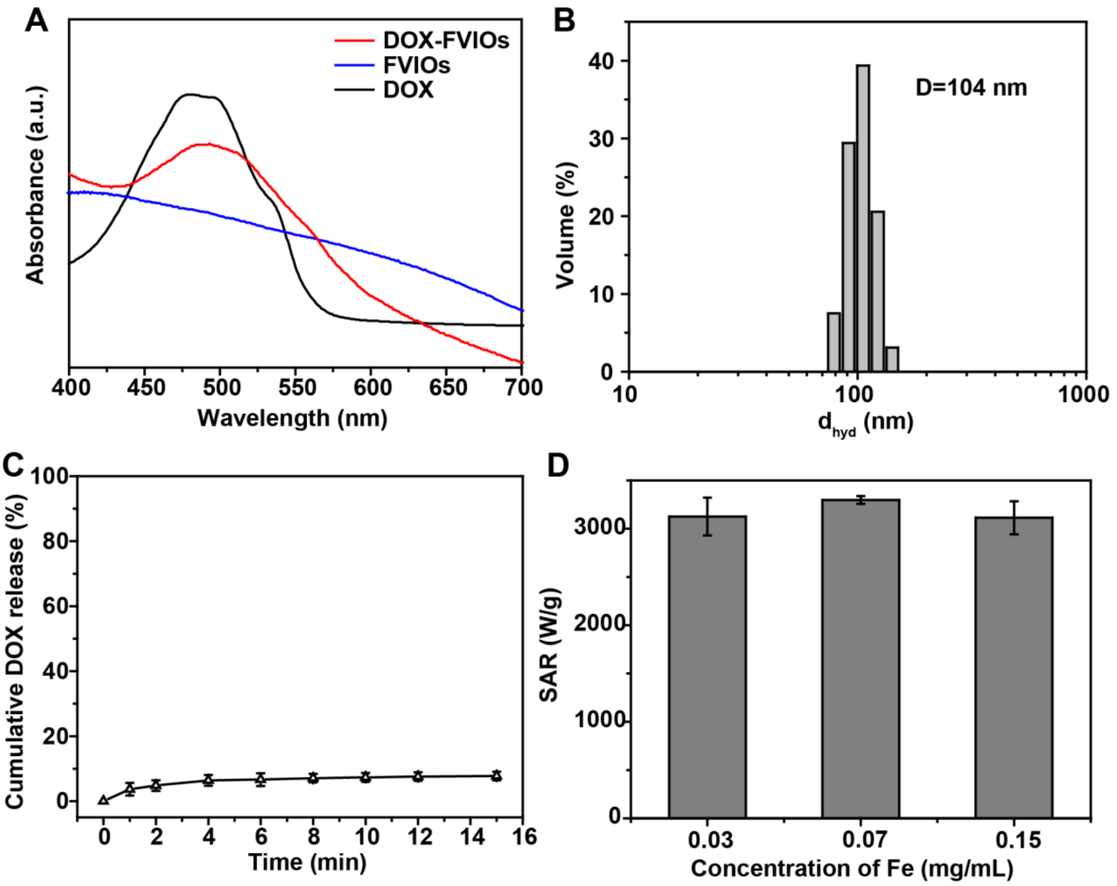

Figure S10. Characterization of DOX-FVIOs. (**A**) UV-Vis absorbance of FVIOs, DOX, and DOX-FVIOs. Experiments were repeated three times. (**B**) Hydrodynamic diameter of DOX-FVIOs in aqueous solution characterized by DLS. Experiments were repeated three times. (**C**) DOX release behavior of DOX-FVIOs during M*f*-EF (360 kHz) exposure. (**D**) SAR values of DOX-FVIOs under M*f*-EF (360 kHz, 300 Oe).

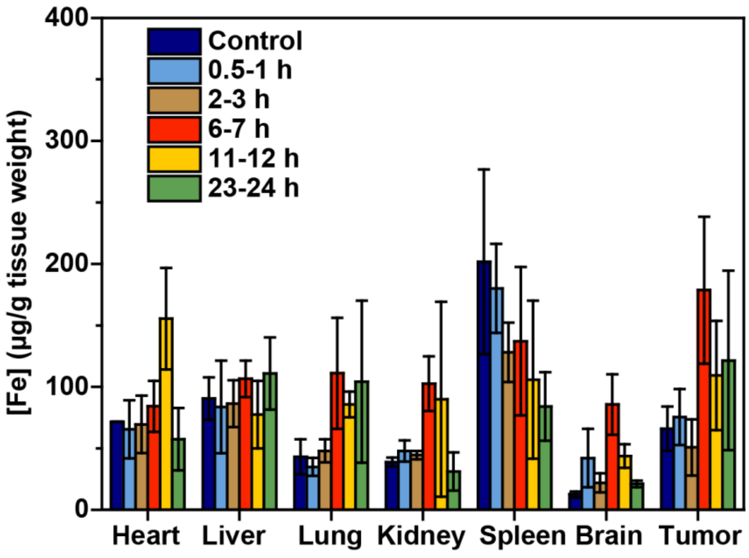

Figure S11. Biodistribution of DOX-PI/FVIOs in mice. MCF-7/ADR tumors-bearing BALB/c nude mice received *i.v.* injections of DOX-PI/FVIOs at an DOX dose of 2.5 mg/kg. Fe concentrations were measured by ICP-MS.

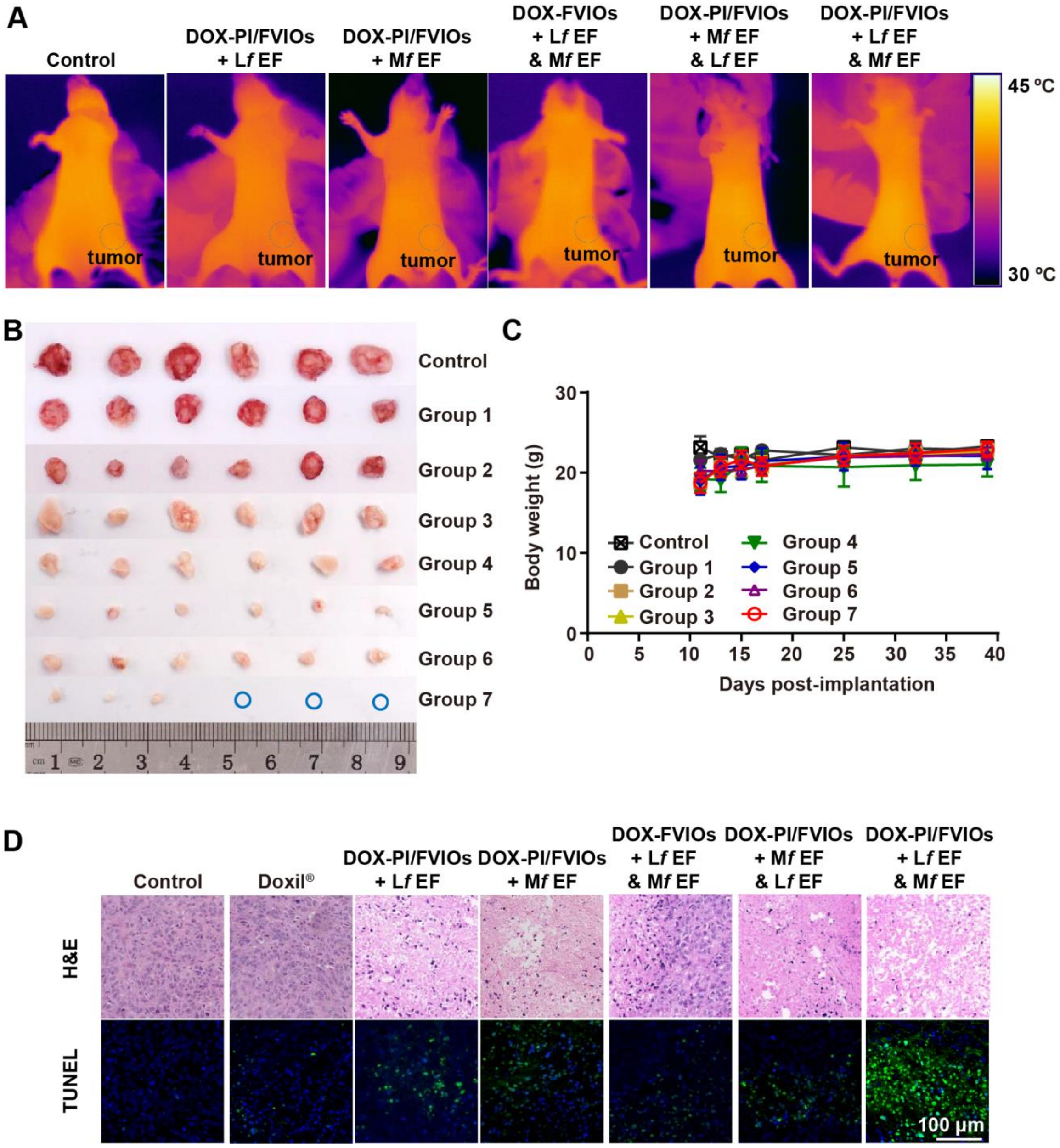

Figure S12. ***In vivo*** **antitumor efficacy in subcutaneous MCF-7/ADR xenograft tumor model.** (**A**) Infrared radiation (IR) thermal images of MCF-7/ADR tumor-bearing mice after various treatments. Tumors are outlined by blue dashed lines. (**B**) Digital photographs of MCF-7/ADR tumor tissues (n = 6) harvested from nude mice at the end of treatment. (**C**) Weights of MCF-7/ADR tumor-bearing mice (n = 6) *versus* days after different treatments. (**D**) Representative images of H&E- and TUNEL-stained sections of MCF-7/ADR tumor tissues for different treatment groups.

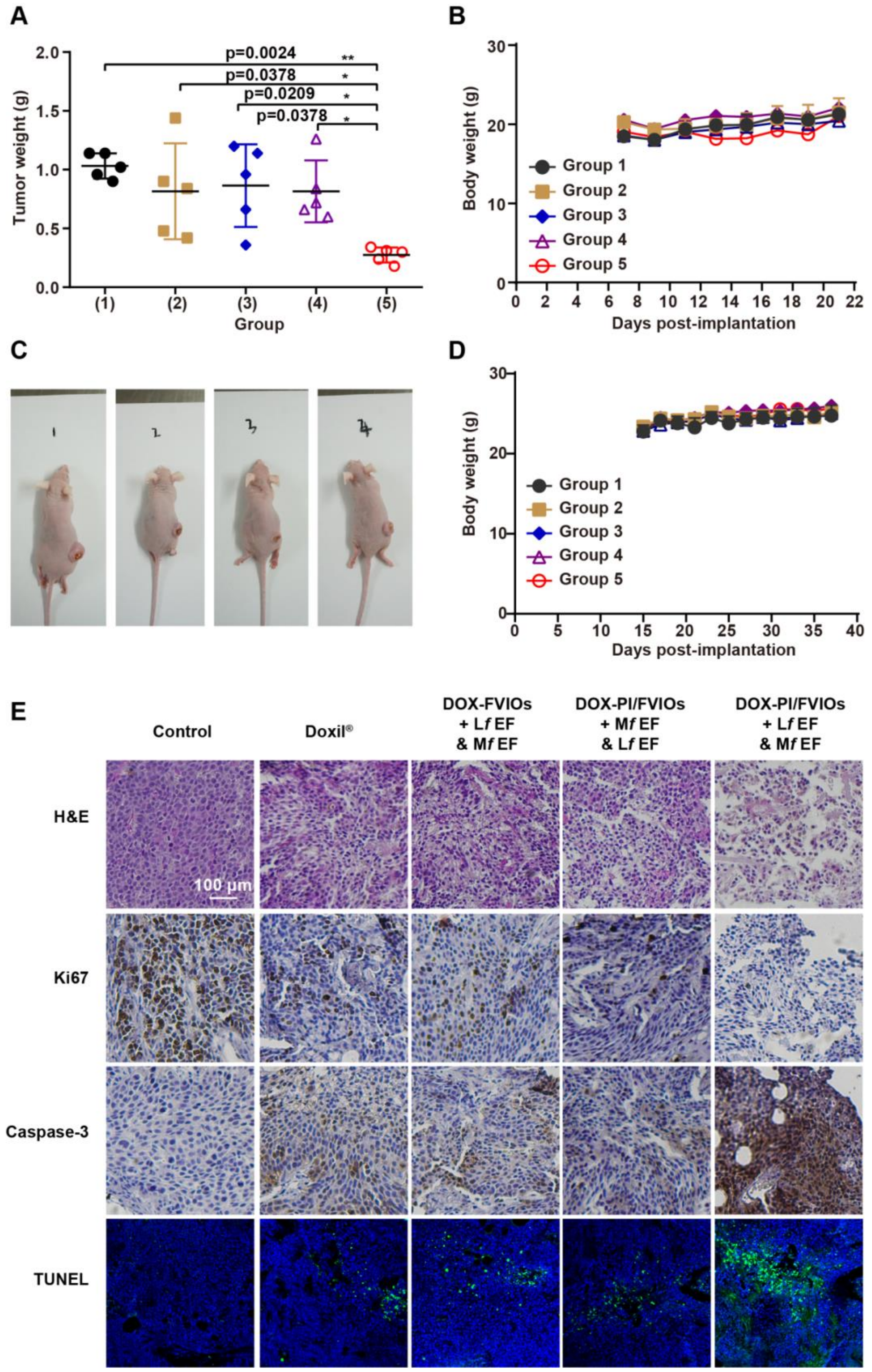

Figure S13. ***In vivo*** **antitumor efficacy in subcutaneous MDA-MB-231 and pancreatic BxPC-3 xenograft tumors.** (**A**) The tumor weights were measured after different treatments. (**B**) Weights of MDA-MB-231 tumor-bearing mice (n = 5) *versus* days after different treatments. (**C**) Images of the pancreatic BxPC-3 xenograft tumors in 4 surviving mice from group 5 at day 80. (**D**) Weights of pancreatic BxPC-3 tumor-bearing mice (n = 5) *versus* days after different treatments. (**E**) Images of tumor sections after H&E staining, immunohistochemical staining of Ki67 and caspase-3, and TUNEL assay.

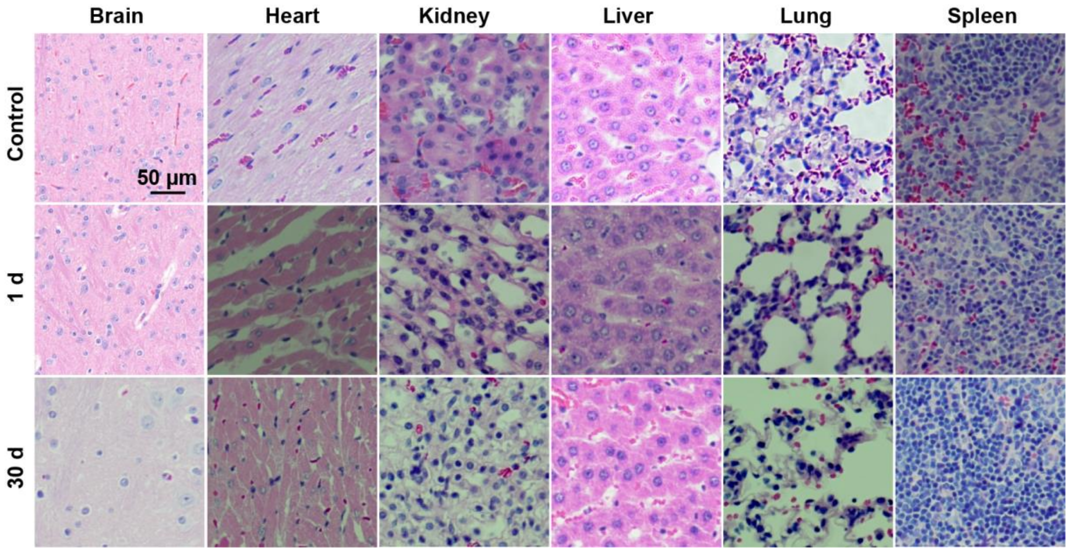

Figure S14. Histological examination of brain, heart, kidney, liver, lung, and spleen tissues by H&E staining. Sprague Dawley (SD) rats (n=3) were injected with saline or DOX-PI/FVIOs ($C_{[Fe]}$ = 5.0 mg/kg) *via* the tail vein. Tissue samples were obtained from the saline (control) group 30 d after injection, and tissue samples were also obtained from DOX-PI/FVIOs group 1 d and 30 d after injection.

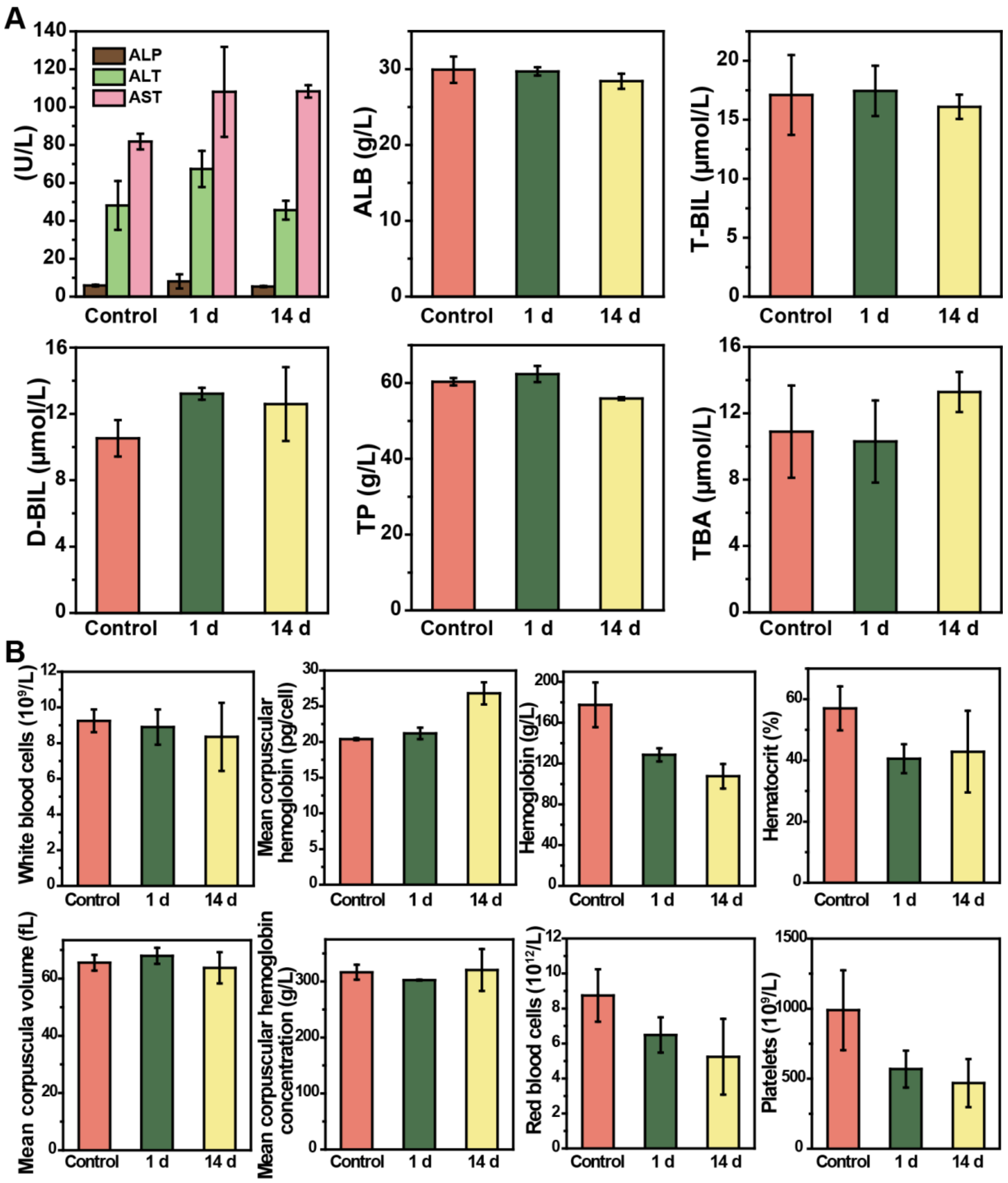

Figure S15. Liver function and blood chemistry of SD rats. SD rats (n = 3) were injected with saline or DOX-PI/FVIOs ($C_{[Fe]}$ = 5.0 mg/kg) *via* tail vein. The mice were euthanized after 1 d or 14 d and the blood samples were collected for analysis. (**A**) Blood biochemistry assessment of liver function markers, including alkaline phosphatase (ALP), alanine aminotransferase (ALT), amino transferase (AST), albumin (ALB), total bilirubin (TBIL), direct bilirubin (DBIL), total protein (TP), and total biliary acid (TBA). (**B**) Routine blood examinations, including white blood cells, mean corpuscular hemoglobin, hemoglobin, hematocrit, mean corpuscular volume, mean corpuscular hemoglobin concentration, red blood cell count, and platelet count.

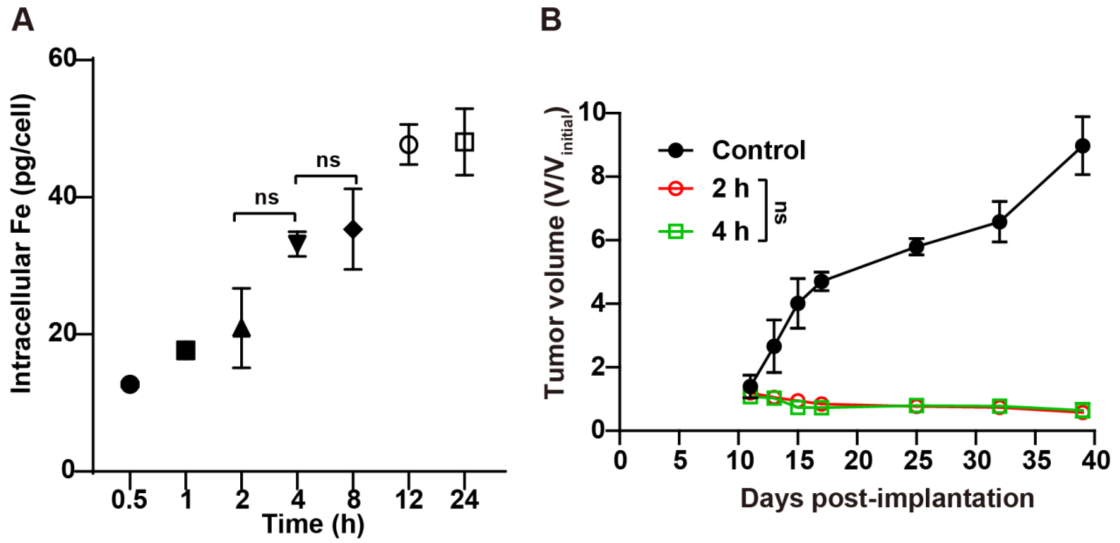

Figure S16. Cellular Fe uptake and *in vivo* antitumor efficacy comparison in subcutaneous MCF-7/ADR xenograft tumor model. (**A**) The intracellular Fe content *versus* incubation time graph for MCF-7/ADR breast cancer cells incubated with PI/FVIOs. The intracellular Fe content increased persistently with incubation time up to 4 h. The highest uptake appeared at approximately 4 h, which there was no significant difference for intracellular Fe content between 2 h and 4 h. (**B**) The tumor volumes were measured for group 8 treatment (DOX-PI/FVIOs plus L*f*-EF and M*f*-EF) with different interval time between L*f*-EF and M*f*-EF. It clearly shows an insignificant difference of antitumor efficacy between two groups.

Table S1. Loading content of DOX with different mass ratios of DOX and PI/FVIOs. The optimal DOX loading content in this study is 26 %.

Mass ratio of DOX and PI/FVIOs ($w_{DOX}/w_{PI/FVIOs}$)	Loading content of DOX ($w_{DOX}/w_{PI/FVIOs}$, %)
0.05	4.19
0.1	8.41
0.2	18
0.4	26.4
0.8	29
1.6	30.1

Movie S1. Movement of PI/FVIO nanovehicles in gelatin.

20 µL PI/FVIOs (1 mg/mL) solution was added to solidified gelatin (with a mass fraction of 2%) in a cuvette, and then a static magnetic field (5000 Oe) was applied for 10 min. The movement of PI/FVIOs was recorded using a video camera.

Movie S2. Enhanced tumor tissue penetration exhibited by DOX-PI/FVIO nanovehicles under L*f*-EF actuation in a 3D reconfigured tumor (80 tumor sections).

Movie S3. The distribution of PI/FVIOs^{FITC} in a 3D reconfigured tumor (80 tumor sections) with L*f*-EF actuation.

Movie S4. The internalization of the nanovehicles for the delivery of DOX (red) into the cell endolysosomes.

Movie S5. After exposure to 360 kHz M*f*-EF for 2-12 min, there is a sharp increase in the DOX fluorescence in the nucleus for DOX-PI/FVIOs.

Movie S6. After exposure to 360 kHz M*f*-EF for 2-12 min, the DOX fluorescence remains in the perinuclear region for DOX-FVIOs.
